# Supplementary material for: Contribution of a Novel B3GLCT Variant to Peters Plus Syndrome Discovered by a Combination of Next-Generation Sequencing and Automated Text Mining
Source: Int J Mol Sci. 2019 Nov 28;20(23):6006. doi: 10.3390/ijms20236006 (PMC6928627; doi:10.3390/ijms20236006)
Supplement: Supplementary file 1 [file ijms-20-06006-s001.zip › Toton-Zuranska et al. Supplement_1.pdf]

```
##fileformat=VCFv4.2
##ALT=<ID=NON_REF,Description="Represents any possible alternative allele
at this location">
##FILTER=<ID=AllGtsFiltered,Description="Site filtered out because all
genotypes are filtered out.">
##FILTER=<ID=AlleleBalance,Description="Heterozygote allele balance below
required threshold.">
##FILTER=<ID=LowQD,Description="Site exhibits QD value below a hard
limit.">
##FILTER=<ID=LowQual,Description="Low quality">
##FILTER=<ID=StrandBias,Description="Site exhibits excessive
allele/strand correlation.">
##FILTER=<ID=VQSRTrancheINDEL99.00to99.90,Description="Truth sensitivity
tranche level for INDEL model at VQS Lod: -3.4882 <= x < -0.7714">
##FILTER=<ID=VQSRTrancheINDEL99.90to100.00+,Description="Truth
sensitivity tranche level for INDEL model at VQS Lod < -13.9132">
##FILTER=<ID=VQSRTrancheINDEL99.90to100.00,Description="Truth sensitivity
tranche level for INDEL model at VQS Lod: -13.9132 <= x < -3.4882">
##FILTER=<ID=VQSRTrancheSNP99.90to100.00+,Description="Truth sensitivity
tranche level for SNP model at VQS Lod < -475.643">
##FILTER=<ID=VQSRTrancheSNP99.90to100.00,Description="Truth sensitivity
tranche level for SNP model at VQS Lod: -475.643 <= x < -3.0101">
##FORMAT=<ID=AD,Number=R,Type=Integer,Description="Allelic depths for the
ref and alt alleles in the order listed">
##FORMAT=<ID=DP,Number=1,Type=Integer,Description="Approximate read depth
(reads with MQ=255 or with bad mates are filtered)">
##FORMAT=<ID=FT,Number=.,Type=String,Description="Genotype filters.">
##FORMAT=<ID=GQ,Number=1,Type=Integer,Description="Genotype Quality">
##FORMAT=<ID=GT,Number=1,Type=String,Description="Genotype">
##FORMAT=<ID=PGT,Number=1,Type=String,Description="Physical phasing
haplotype information, describing how the alternate alleles are phased in
relation to one another">
##FORMAT=<ID=PID,Number=1,Type=String,Description="Physical phasing ID
information, where each unique ID within a given sample (but not across
samples) connects records within a phasing group">
##FORMAT=<ID=PL,Number=G,Type=Integer,Description="Normalized, Phred-
scaled likelihoods for genotypes as defined in the VCF specification">
##FORMAT=<ID=RGQ,Number=1,Type=Integer,Description="Unconditional
reference genotype confidence, encoded as a phred quality -10*log10
p(genotype call is wrong)">
##FORMAT=<ID=SB,Number=4,Type=Integer,Description="Per-sample component
statistics which comprise the Fisher's Exact Test to detect strand
bias.">
##INFO=<ID=AC,Number=A,Type=Integer,Description="Allele count in
genotypes, for each ALT allele, in the same order as listed">
##INFO=<ID=AF,Number=A,Type=Float,Description="Allele Frequency, for each
ALT allele, in the same order as listed">
##INFO=<ID=AN,Number=1,Type=Integer,Description="Total number of alleles
in called genotypes">
##INFO=<ID=BaseQRankSum,Number=1,Type=Float,Description="Z-score from
Wilcoxon rank sum test of Alt Vs. Ref base qualities">
##INFO=<ID=ClippingRankSum,Number=1,Type=Float,Description="Z-score From
Wilcoxon rank sum test of Alt vs. Ref number of hard clipped bases">
##INFO=<ID=DB,Number=0,Type=Flag,Description="dbSNP Membership">
##INFO=<ID=DP,Number=1,Type=Integer,Description="Approximate read depth;
some reads may have been filtered">
##INFO=<ID=DS,Number=0,Type=Flag,Description="Were any of the samples
downsampled?">
```

```
##INFO=<ID=END,Number=1,Type=Integer,Description="Stop position of the
interval">
##INFO=<ID=ExcessHet,Number=1,Type=Float,Description="Phred-scaled p-
value for exact test of excess heterozygosity">
##INFO=<ID=FS,Number=1,Type=Float,Description="Phred-scaled p-value using
Fisher's exact test to detect strand bias">
##INFO=<ID=HaplotypeScore,Number=1,Type=Float,Description="Consistency of
the site with at most two segregating haplotypes">
##INFO=<ID=InbreedingCoeff,Number=1,Type=Float,Description="Inbreeding
coefficient as estimated from the genotype likelihoods per-sample when
compared against the Hardy-Weinberg expectation">
##INFO=<ID=MLEAC,Number=A,Type=Integer,Description="Maximum likelihood
expectation (MLE) for the allele counts (not necessarily the same as the
AC), for each ALT allele, in the same order as listed">
##INFO=<ID=MLEAF,Number=A,Type=Float,Description="Maximum likelihood
expectation (MLE) for the allele frequency (not necessarily the same as
the AF), for each ALT allele, in the same order as listed">
##INFO=<ID=MQ,Number=1,Type=Float,Description="RMS Mapping Quality">
##INFO=<ID=MQRankSum,Number=1,Type=Float,Description="Z-score From
Wilcoxon rank sum test of Alt vs. Ref read mapping qualities">
##INFO=<ID=NEGATIVE_TRAIN_SITE,Number=0,Type=Flag,Description="This
variant was used to build the negative training set of bad variants">
##INFO=<ID=POSITIVE_TRAIN_SITE,Number=0,Type=Flag,Description="This
variant was used to build the positive training set of good variants">
##INFO=<ID=QD,Number=1,Type=Float,Description="Variant Confidence/Quality
by Depth">
##INFO=<ID=RAW_MQ,Number=1,Type=Float,Description="Raw data for RMS
Mapping Quality">
##INFO=<ID=ReadPosRankSum,Number=1,Type=Float,Description="Z-score from
Wilcoxon rank sum test of Alt vs. Ref read position bias">
##INFO=<ID=SOR,Number=1,Type=Float,Description="Symmetric Odds Ratio of
2x2 contingency table to detect strand bias">
##INFO=<ID=VQSLOD,Number=1,Type=Float,Description="Log odds of being a
true variant versus being false under the trained gaussian mixture
model">
##INFO=<ID=culprit,Number=1,Type=String,Description="The annotation which
was the worst performing in the Gaussian mixture model, likely the reason
why the variant was filtered out">
##contig=<ID=chrM,length=16571,assembly=hg19>
##contig=<ID=chr1,length=249250621,assembly=hg19>
##contig=<ID=chr2,length=243199373,assembly=hg19>
##contig=<ID=chr3,length=198022430,assembly=hg19>
##contig=<ID=chr4,length=191154276,assembly=hg19>
##contig=<ID=chr5,length=180915260,assembly=hg19>
##contig=<ID=chr6,length=171115067,assembly=hg19>
##contig=<ID=chr7,length=159138663,assembly=hg19>
##contig=<ID=chr8,length=146364022,assembly=hg19>
##contig=<ID=chr9,length=141213431,assembly=hg19>
##contig=<ID=chr10,length=135534747,assembly=hg19>
##contig=<ID=chr11,length=135006516,assembly=hg19>
##contig=<ID=chr12,length=133851895,assembly=hg19>
##contig=<ID=chr13,length=115169878,assembly=hg19>
##contig=<ID=chr14,length=107349540,assembly=hg19>
##contig=<ID=chr15,length=102531392,assembly=hg19>
##contig=<ID=chr16,length=90354753,assembly=hg19>
##contig=<ID=chr17,length=81195210,assembly=hg19>
##contig=<ID=chr18,length=78077248,assembly=hg19>
##contig=<ID=chr19,length=59128983,assembly=hg19>
```

```
##contig=<ID=chr20,length=63025520,assembly=hg19>
##contig=<ID=chr21,length=48129895,assembly=hg19>
##contig=<ID=chr22,length=51304566,assembly=hg19>
##contig=<ID=chrX,length=155270560,assembly=hg19>
##contig=<ID=chrY,length=59373566,assembly=hg19>
##contig=<ID=chr1_gl000191_random,length=106433,assembly=hg19>
##contig=<ID=chr1_gl000192_random,length=547496,assembly=hg19>
##contig=<ID=chr4_ctg9_hap1,length=590426,assembly=hg19>
##contig=<ID=chr4_gl000193_random,length=189789,assembly=hg19>
##contig=<ID=chr4_gl000194_random,length=191469,assembly=hg19>
##contig=<ID=chr6_apd_hap1,length=4622290,assembly=hg19>
##contig=<ID=chr6_cox_hap2,length=4795371,assembly=hg19>
##contig=<ID=chr6_dbb_hap3,length=4610396,assembly=hg19>
##contig=<ID=chr6_mann_hap4,length=4683263,assembly=hg19>
##contig=<ID=chr6_mcf_hap5,length=4833398,assembly=hg19>
##contig=<ID=chr6_qbl_hap6,length=4611984,assembly=hg19>
##contig=<ID=chr6_ssto_hap7,length=4928567,assembly=hg19>
##contig=<ID=chr7_gl000195_random,length=182896,assembly=hg19>
##contig=<ID=chr8_gl000196_random,length=38914,assembly=hg19>
##contig=<ID=chr8_gl000197_random,length=37175,assembly=hg19>
##contig=<ID=chr9_gl000198_random,length=90085,assembly=hg19>
##contig=<ID=chr9_gl000199_random,length=169874,assembly=hg19>
##contig=<ID=chr9_gl000200_random,length=187035,assembly=hg19>
##contig=<ID=chr9_gl000201_random,length=36148,assembly=hg19>
##contig=<ID=chr11_gl000202_random,length=40103,assembly=hg19>
##contig=<ID=chr17_ctg5_hap1,length=1680828,assembly=hg19>
##contig=<ID=chr17_gl000203_random,length=37498,assembly=hg19>
##contig=<ID=chr17_gl000204_random,length=81310,assembly=hg19>
##contig=<ID=chr17_gl000205_random,length=174588,assembly=hg19>
##contig=<ID=chr17_gl000206_random,length=41001,assembly=hg19>
##contig=<ID=chr18_gl000207_random,length=4262,assembly=hg19>
##contig=<ID=chr19_gl000208_random,length=92689,assembly=hg19>
##contig=<ID=chr19_gl000209_random,length=159169,assembly=hg19>
##contig=<ID=chr21_gl000210_random,length=27682,assembly=hg19>
##contig=<ID=chrUn_gl000211,length=166566,assembly=hg19>
##contig=<ID=chrUn_gl000212,length=186858,assembly=hg19>
##contig=<ID=chrUn_gl000213,length=164239,assembly=hg19>
##contig=<ID=chrUn_gl000214,length=137718,assembly=hg19>
##contig=<ID=chrUn_gl000215,length=172545,assembly=hg19>
##contig=<ID=chrUn_gl000216,length=172294,assembly=hg19>
##contig=<ID=chrUn_gl000217,length=172149,assembly=hg19>
##contig=<ID=chrUn_gl000218,length=161147,assembly=hg19>
##contig=<ID=chrUn_gl000219,length=179198,assembly=hg19>
##contig=<ID=chrUn_gl000220,length=161802,assembly=hg19>
##contig=<ID=chrUn_gl000221,length=155397,assembly=hg19>
##contig=<ID=chrUn_gl000222,length=186861,assembly=hg19>
##contig=<ID=chrUn_gl000223,length=180455,assembly=hg19>
##contig=<ID=chrUn_gl000224,length=179693,assembly=hg19>
##contig=<ID=chrUn_gl000225,length=211173,assembly=hg19>
##contig=<ID=chrUn_gl000226,length=15008,assembly=hg19>
##contig=<ID=chrUn_gl000227,length=128374,assembly=hg19>
##contig=<ID=chrUn_gl000228,length=129120,assembly=hg19>
##contig=<ID=chrUn_gl000229,length=19913,assembly=hg19>
##contig=<ID=chrUn_gl000230,length=43691,assembly=hg19>
##contig=<ID=chrUn_gl000231,length=27386,assembly=hg19>
##contig=<ID=chrUn_gl000232,length=40652,assembly=hg19>
##contig=<ID=chrUn_gl000233,length=45941,assembly=hg19>
##contig=<ID=chrUn_gl000234,length=40531,assembly=hg19>
```

```
##contig=<ID=chrUn_gl000235,length=34474,assembly=hg19>
##contig=<ID=chrUn_gl000236,length=41934,assembly=hg19>
##contig=<ID=chrUn_gl000237,length=45867,assembly=hg19>
##contig=<ID=chrUn_gl000238,length=39939,assembly=hg19>
##contig=<ID=chrUn_gl000239,length=33824,assembly=hg19>
##contig=<ID=chrUn_gl000240,length=41933,assembly=hg19>
##contig=<ID=chrUn_gl000241,length=42152,assembly=hg19>
##contig=<ID=chrUn_gl000242,length=43523,assembly=hg19>
##contig=<ID=chrUn_gl000243,length=43341,assembly=hg19>
##contig=<ID=chrUn_gl000244,length=39929,assembly=hg19>
##contig=<ID=chrUn_gl000245,length=36651,assembly=hg19>
##contig=<ID=chrUn_gl000246,length=38154,assembly=hg19>
##contig=<ID=chrUn_gl000247,length=36422,assembly=hg19>
##contig=<ID=chrUn_gl000248,length=39786,assembly=hg19>
##contig=<ID=chrUn_gl000249,length=38502,assembly=hg19>
##reference=file:///resources/hg19/ucsc.hg19.fasta
##source=SelectVariants
#CHROM      POS   ID    REF   ALT   QUAL  FILTER      INFO  FORMAT
      s42.ABL7Y
chr1  14099763    .     C     T     5398.21     PASS
      AC=2;AF=1.00;AN=2;BaseQRankSum=-
1.141e+00;ClippingRankSum=0.00;DP=10;ExcessHet=5.4021;FS=1.118;Inbreeding
Coeff=-
0.0998;MQ=60.00;MQRankSum=0.00;POSITIVE_TRAIN_SITE;QD=12.44;ReadPosRankSu
m=0.087;SOR=0.598;VQSLOD=15.36;culprit=MQRankSum   GT:AD:DP:GQ:PL
      1/1:0,10:10:30:313,30,0
chr1  15820576    .     T     A     13755.72    PASS
      AC=2;AF=1.00;AN=2;BaseQRankSum=1.66;ClippingRankSum=0.00;DP=23;Exce
ssHet=3.3693;FS=0.000;InbreedingCoeff=-
0.0444;MQ=59.85;MQRankSum=0.00;POSITIVE_TRAIN_SITE;QD=20.87;ReadPosRankSu
m=0.054;SOR=0.713;VQSLOD=7.91;culprit=MQRankSum    GT:AD:DP:GQ:PL
      1/1:0,23:23:68:734,68,0
chr1  15821673    .     C     T     7288.17     PASS
      AC=2;AF=1.00;AN=2;BaseQRankSum=-9.650e-
01;ClippingRankSum=0.00;DP=20;ExcessHet=3.5546;FS=12.105;InbreedingCoeff=
-
0.0328;MQ=60.00;MQRankSum=0.00;POSITIVE_TRAIN_SITE;QD=12.57;ReadPosRankSu
m=0.393;SOR=0.241;VQSLOD=9.60;culprit=MQRankSum    GT:AD:DP:GQ:PL
      1/1:0,20:20:59:620,59,0
chr1  15834297    .     G     A     27187.53    PASS
      AC=2;AF=1.00;AN=2;BaseQRankSum=-
1.858e+00;ClippingRankSum=0.00;DP=48;ExcessHet=2.1346;FS=3.272;Inbreeding
Coeff=0.0330;MQ=60.00;MQRankSum=0.00;POSITIVE_TRAIN_SITE;QD=18.00;ReadPos
RankSum=0.079;SOR=0.472;VQSLOD=14.33;culprit=MQRankSum  GT:AD:DP:GQ:PL
      1/1:0,48:48:99:1402,144,0
chr1  15834491    .     G     A     15837.57    PASS
      AC=2;AF=1.00;AN=2;BaseQRankSum=0.786;ClippingRankSum=0.00;DP=19;Exc
essHet=2.1346;FS=3.362;InbreedingCoeff=0.0328;MQ=60.00;MQRankSum=0.00;POS
ITIVE_TRAIN_SITE;QD=19.72;ReadPosRankSum=-5.620e-
01;SOR=0.458;VQSLOD=13.82;culprit=MQRankSum  GT:AD:DP:GQ:PL
      1/1:0,19:19:57:661,57,0
chr1  15850792    .     GC    G     5874.37     PASS
      AC=2;AF=1.00;AN=2;BaseQRankSum=0.364;ClippingRankSum=0.00;DP=17;Exc
essHet=3.9220;FS=7.086;InbreedingCoeff=-
0.0655;MQ=60.00;MQRankSum=0.00;QD=16.14;ReadPosRankSum=0.124;SOR=0.309;VQ
SLOD=5.33;culprit=MQRankSum GT:AD:DP:GQ:PL   1/1:0,17:17:51:559,51,0
chr1  16891485    .     C     T     56532.89    PASS
      AC=2;AF=1.00;AN=2;BaseQRankSum=-
```

2.771e+00;ClippingRankSum=0.00;DP=49;ExcessHet=45.0187;FS=1.051;InbreedingCoeff=-0.6459;MQ=46.81;MQRankSum=-
2.673e+00;NEGATIVE_TRAIN_SITE;QD=21.00;ReadPosRankSum=0.214;SOR=0.838;VQSLOD=-3.295e+00;culprit=MQRankSum GT:AD:DP:GQ:PL
1/1:2,47:49:99:1430,101,0
chr1 43650741 . T C 47929.53 PASS
AC=2;AF=1.00;AN=2;BaseQRankSum=2.35;ClippingRankSum=0.00;DP=75;ExcessHet=8.6905;FS=0.574;InbreedingCoeff=-
0.1843;MQ=60.00;MQRankSum=0.00;POSITIVE_TRAIN_SITE;QD=16.76;ReadPosRankSum=0.244;SOR=0.784;VQSLOD=14.01;culprit=MQRankSum GT:AD:DP:GQ:PL
1/1:0,75:75:99:2424,225,0
chr1 53793510 . CTG C 356.46 PASS
AC=1;AF=0.500;AN=2;BaseQRankSum=0.253;ClippingRankSum=0.00;DP=10;ExcessHet=6.6726;FS=0.000;InbreedingCoeff=-
0.1768;MQ=66.77;MQRankSum=1.38;QD=2.36;ReadPosRankSum=0.00;SOR=0.681;VQSLOD=-7.500e-03;culprit=MQRankSum GT:AD:DP:GQ:PGT:PID:PL
0/1:7,3:10:85:0|1:53793510_CTG_C:85,0,389
chr1 53793513 . CA C 361.80 PASS
AC=1;AF=0.500;AN=2;BaseQRankSum=0.00;ClippingRankSum=0.00;DP=10;ExcessHet=9.6196;FS=0.000;InbreedingCoeff=-
0.2184;MQ=66.80;MQRankSum=1.38;QD=2.41;ReadPosRankSum=-2.720e-
01;SOR=0.666;VQSLOD=-3.980e-02;culprit=MQRankSum GT:AD:DP:GQ:PGT:PID:PL
0/1:7,3:10:85:0|1:53793510_CTG_C:85,0,389
chr1 63871881 . A G 7474.90 PASS
AC=2;AF=1.00;AN=2;BaseQRankSum=2.66;ClippingRankSum=0.00;DP=16;ExcessHet=0.6874;FS=10.441;InbreedingCoeff=0.1464;MQ=59.95;MQRankSum=0.00;POSITIVE_TRAIN_SITE;QD=19.72;ReadPosRankSum=0.208;SOR=1.666;VQSLOD=8.69;culprit=MQRankSum GT:AD:DP:GQ:PL 1/1:0,16:16:48:559,48,0
chr1 63872871 . A G 11263.02 PASS
AC=2;AF=1.00;AN=2;BaseQRankSum=0.094;ClippingRankSum=0.00;DP=14;ExcessHet=0.2172;FS=0.000;InbreedingCoeff=0.2492;MQ=59.99;MQRankSum=0.00;POSITIVE_TRAIN_SITE;QD=16.74;ReadPosRankSum=-9.000e-
03;SOR=0.718;VQSLOD=11.15;culprit=MQRankSum GT:AD:DP:GQ:PL
1/1:0,14:14:42:377,42,0
chr1 86906114 . T C 8733.59 PASS
AC=2;AF=1.00;AN=2;BaseQRankSum=2.40;ClippingRankSum=0.00;DP=10;ExcessHet=7.7774;FS=4.952;InbreedingCoeff=-
0.1619;MQ=60.00;MQRankSum=0.00;POSITIVE_TRAIN_SITE;QD=18.19;ReadPosRankSum=0.00;SOR=1.292;VQSLOD=12.88;culprit=MQRankSum GT:AD:DP:GQ:PL
1/1:0,10:10:30:363,30,0
chr1 92195601 . G A 14056.64 PASS
AC=2;AF=1.00;AN=2;BaseQRankSum=-
1.157e+00;ClippingRankSum=0.00;DP=15;ExcessHet=1.7034;FS=4.074;InbreedingCoeff=0.0581;MQ=59.98;MQRankSum=0.00;POSITIVE_TRAIN_SITE;QD=18.72;ReadPosRankSum=-1.570e-01;SOR=0.410;VQSLOD=8.52;culprit=MQRankSum
GT:AD:DP:GQ:PL 1/1:0,15:15:45:491,45,0
chr1 111773273 . T C 2226 PASS
AC=2;AF=1.00;AN=2;BaseQRankSum=0.619;ClippingRankSum=0.00;DP=10;ExcessHet=0.2460;FS=2.810;InbreedingCoeff=0.1920;MQ=60.00;MQRankSum=0.00;POSITIVE_TRAIN_SITE;QD=16.74;ReadPosRankSum=-3.010e-
01;SOR=0.239;VQSLOD=12.62;culprit=MQRankSum GT:AD:DP:GQ:PL
1/1:0,10:10:30:337,30,0
chr1 160093927 . T A 6315.85 PASS
AC=2;AF=1.00;AN=2;BaseQRankSum=-5.590e-
01;ClippingRankSum=0.00;DP=14;ExcessHet=1.3050;FS=1.763;InbreedingCoeff=0.0884;MQ=59.96;MQRankSum=0.00;POSITIVE_TRAIN_SITE;QD=14.13;ReadPosRankSum=0.160;SOR=0.796;VQSLOD=9.96;culprit=MQRankSum GT:AD:DP:GQ:PL
1/1:0,14:14:42:415,42,0

chr1 209812063 . T C 21973.75 PASS
AC=2;AF=1.00;AN=2;BaseQRankSum=0.971;ClippingRankSum=0.00;DP=30;ExcessHet=1.3050;FS=0.628;InbreedingCoeff=0.0905;MQ=60.00;MQRankSum=0.00;POSITIVE_TRAIN_SITE;QD=20.31;ReadPosRankSum=-4.360e-01;SOR=0.592;VQSLOD=13.74;culprit=MQRankSum GT:AD:DP:GQ:PL
1/1:0,30:30:88:789,88,0
chr1 215849044 . C G 2735.98 PASS
AC=2;AF=1.00;AN=2;BaseQRankSum=-1.615e+00;ClippingRankSum=0.00;DP=11;ExcessHet=1.4500;FS=2.464;InbreedingCoeff=0.0324;MQ=59.87;MQRankSum=0.00;POSITIVE_TRAIN_SITE;QD=12.05;ReadPosRankSum=0.615;SOR=0.888;VQSLOD=7.52;culprit=MQRankSum GT:AD:DP:GQ:PL
1/1:0,11:11:33:334,33,0
chr1 222835522 . AT A 311.91 PASS
AC=1;AF=0.500;AN=2;BaseQRankSum=-6.400e-02;ClippingRankSum=0.00;DP=52;ExcessHet=6.0235;FS=0.000;InbreedingCoeff=-0.1284;MQ=101.57;MQRankSum=0.00;QD=0.80;ReadPosRankSum=0.121;SOR=0.644;VQSLOD=4.93;culprit=QD GT:AD:DP:GQ:PL 0/1:44,8:52:54:54,0,1094
chr1 228497053 . A G 16268.28 PASS
AC=2;AF=1.00;AN=2;BaseQRankSum=1.35;ClippingRankSum=0.00;DP=12;ExcessHet=0.1576;FS=0.652;InbreedingCoeff=0.2216;MQ=60.00;MQRankSum=0.00;POSITIVE_TRAIN_SITE;QD=22.88;ReadPosRankSum=0.066;SOR=0.624;VQSLOD=10.78;culprit=MQRankSum GT:AD:DP:GQ:PL 1/1:0,12:12:36:414,36,0
chr1 228523618 . G A 16672.50 PASS
AC=2;AF=1.00;AN=2;BaseQRankSum=-5.740e-01;ClippingRankSum=0.00;DP=38;ExcessHet=0.0751;FS=1.397;InbreedingCoeff=0.3160;MQ=60.00;MQRankSum=0.00;POSITIVE_TRAIN_SITE;QD=20.06;ReadPosRankSum=0.520;SOR=0.844;VQSLOD=9.71;culprit=MQRankSum GT:AD:DP:GQ:PL
1/1:0,38:38:99:1055,113,0
chr1 228525898 . C G 25895.94 PASS
AC=2;AF=1.00;AN=2;BaseQRankSum=0.113;ClippingRankSum=0.00;DP=37;ExcessHet=0.0661;FS=0.000;InbreedingCoeff=0.3017;MQ=60.00;MQRankSum=0.00;POSITIVE_TRAIN_SITE;QD=22.96;ReadPosRankSum=-5.320e-01;SOR=0.707;VQSLOD=10.51;culprit=MQRankSum GT:AD:DP:GQ:PL
1/1:0,37:37:99:1240,111,0
chr1 242016590 . C T 5704.03 PASS
AC=2;AF=1.00;AN=2;BaseQRankSum=-1.265e+00;ClippingRankSum=0.00;DP=20;ExcessHet=0.5554;FS=3.489;InbreedingCoeff=0.1661;MQ=59.94;MQRankSum=0.00;POSITIVE_TRAIN_SITE;QD=15.50;ReadPosRankSum=-6.080e-01;SOR=0.908;VQSLOD=7.72;culprit=MQRankSum
GT:AD:DP:GQ:PL 1/1:0,20:20:60:606,60,0
chr2 30977073 . G A 24521.51 PASS
AC=2;AF=1.00;AN=2;BaseQRankSum=1.84;ClippingRankSum=0.00;DP=18;ExcessHet=2.4407;FS=4.582;InbreedingCoeff=0.0052;MQ=60.00;MQRankSum=0.00;POSITIVE_TRAIN_SITE;QD=24.55;ReadPosRankSum=0.389;SOR=1.323;VQSLOD=11.74;culprit=MQRankSum GT:AD:DP:GQ:PL 1/1:0,18:18:54:540,54,0
chr2 43799070 . C T 26613.75 PASS
AC=2;AF=1.00;AN=2;BaseQRankSum=-2.945e+00;ClippingRankSum=0.00;DP=12;ExcessHet=4.7290;FS=11.992;InbreedingCoeff=-0.0820;MQ=60.00;MQRankSum=0.00;POSITIVE_TRAIN_SITE;QD=22.27;ReadPosRankSum=-7.800e-02;SOR=0.137;VQSLOD=9.48;culprit=MQRankSum GT:AD:DP:GQ:PL
1/1:0,12:12:36:392,36,0
chr2 44071833 . A C 5135.01 PASS
AC=2;AF=1.00;AN=2;BaseQRankSum=2.14;ClippingRankSum=0.00;DP=13;ExcessHet=5.4671;FS=15.793;InbreedingCoeff=-0.1529;MQ=59.54;MQRankSum=0.00;POSITIVE_TRAIN_SITE;QD=15.95;ReadPosRankSum=-9.440e-01;SOR=2.076;VQSLOD=6.28;culprit=MQRankSum GT:AD:DP:GQ:PL
1/1:0,13:13:39:439,39,0

chr2 44071852 . G C 3123.28 PASS
AC=2;AF=1.00;AN=2;BaseQRankSum=1.28;ClippingRankSum=0.00;DP=10;ExcessHet=6.4630;FS=11.578;InbreedingCoeff=-0.1799;MQ=59.75;MQRankSum=0.00;POSITIVE_TRAIN_SITE;QD=13.46;ReadPosRankSum=-1.800e-01;SOR=2.017;VQSLOD=7.24;culprit=MQRankSum GT:AD:DP:GQ:PL
1/1:0,10:10:30:365,30,0
chr2 44152406 . G T 8654.04 PASS
AC=2;AF=1.00;AN=2;BaseQRankSum=-1.939e+00;ClippingRankSum=0.00;DP=11;ExcessHet=4.4460;FS=0.678;InbreedingCoeff=-0.0721;MQ=60.00;MQRankSum=0.00;POSITIVE_TRAIN_SITE;QD=16.24;ReadPosRankSum=0.072;SOR=0.785;VQSLOD=15.29;culprit=MQRankSum GT:AD:DP:GQ:PL
1/1:0,11:11:33:353,33,0
chr2 50755683 . GCT G 234.98 PASS
AC=1;AF=0.500;AN=2;BaseQRankSum=0.383;ClippingRankSum=0.00;DP=20;ExcessHet=3.7639;FS=0.000;InbreedingCoeff=-0.0642;MQ=60.00;MQRankSum=0.00;QD=1.17;ReadPosRankSum=0.130;SOR=0.622;VQSLOD=4.85;culprit=QD GT:AD:DP:GQ:PL 0/1:15,5:20:99:133,0,515
chr2 113819614 . T C 13029.73 PASS
AC=2;AF=1.00;AN=2;BaseQRankSum=0.742;ClippingRankSum=0.00;DP=10;ExcessHet=0.1013;FS=0.000;InbreedingCoeff=0.2642;MQ=60.00;MQRankSum=0.00;POSITIVE_TRAIN_SITE;QD=22.01;ReadPosRankSum=-1.500e-02;SOR=0.650;VQSLOD=10.98;culprit=MQRankSum GT:AD:DP:GQ:PL
1/1:0,10:10:30:332,30,0
chr2 179406044 . C T 212.20 PASS
AC=1;AF=0.500;AN=2;BaseQRankSum=-2.420e-01;ClippingRankSum=0.00;DP=19;ExcessHet=3.1627;FS=2.062;InbreedingCoeff=-0.0443;MQ=60.00;MQRankSum=0.00;POSITIVE_TRAIN_SITE;QD=7.07;ReadPosRankSum=1.50;SOR=1.609;VQSLOD=13.80;culprit=MQRankSum GT:AD:DP:GQ:PL
0/1:14,5:19:88:88,0,346
chr2 179474228 . C A 910.26 PASS
AC=1;AF=0.500;AN=2;BaseQRankSum=-3.060e-01;ClippingRankSum=0.00;DP=47;ExcessHet=3.0605;FS=0.832;InbreedingCoeff=-0.0236;MQ=60.00;MQRankSum=0.00;QD=10.58;ReadPosRankSum=1.62;SOR=0.525;VQSLOD=13.90;culprit=MQRankSum GT:AD:DP:GQ:PL 0/1:26,21:47:99:455,0,612
chr2 179548700 . T A 13.92 PASS
AC=1;AF=0.500;AN=2;BaseQRankSum=-9.210e-01;ClippingRankSum=0.00;DP=8;ExcessHet=3.0103;FS=0.000;InbreedingCoeff=-0.0172;MQ=60.00;MQRankSum=0.00;QD=1.74;ReadPosRankSum=-5.240e-01;SOR=0.693;VQSLOD=12.19;culprit=MQRankSum GT:AD:DP:GQ:PL
0/1:6,2:8:53:53,0,174
chr2 179612883 . A G 7659.69 PASS
AC=1;AF=0.500;AN=2;BaseQRankSum=2.68;ClippingRankSum=0.00;DP=171;ExcessHet=3.1627;FS=0.529;InbreedingCoeff=-0.0353;MQ=60.00;MQRankSum=0.00;POSITIVE_TRAIN_SITE;QD=12.20;ReadPosRankSum=0.034;SOR=0.767;VQSLOD=15.41;culprit=MQRankSum GT:AD:DP:GQ:PL
0/1:91,80:171:99:1914,0,2266
chr2 208973074 . G A 45863.38 PASS
AC=2;AF=1.00;AN=2;BaseQRankSum=-5.650e-01;ClippingRankSum=0.00;DP=144;ExcessHet=3.5264;FS=0.537;InbreedingCoeff=-0.0342;MQ=59.70;MQRankSum=0.00;POSITIVE_TRAIN_SITE;QD=13.99;ReadPosRankSum=0.349;SOR=0.735;VQSLOD=7.19;culprit=MQRankSum GT:AD:DP:GQ:PL
1/1:0,144:144:99:3922,431,0
chr2 208973252 . C T 25391.38 PASS
AC=2;AF=1.00;AN=2;BaseQRankSum=1.36;ClippingRankSum=0.00;DP=48;ExcessHet=3.5264;FS=0.542;InbreedingCoeff=-0.0342;MQ=59.91;MQRankSum=0.00;POSITIVE_TRAIN_SITE;QD=14.69;ReadPosRankSu

m=0.803;SOR=0.619;VQSLOD=7.59;culprit=MQRankSum GT:AD:DP:GQ:PL
1/1:0,48:48:99:1297,143,0
chr2 208976955 . A C 10591.69 PASS
AC=2;AF=1.00;AN=2;BaseQRankSum=1.60;ClippingRankSum=0.00;DP=16;ExcessHet=2.3742;FS=3.794;InbreedingCoeff=-0.0030;MQ=56.37;MQRankSum=0.579;NEGATIVE_TRAIN_SITE;POSITIVE_TRAIN_SITE;QD=19.83;ReadPosRankSum=0.205;SOR=1.219;VQSLOD=0.169;culprit=MQRankSum
GT:AD:DP:GQ:PL 1/1:0,16:16:48:531,48,0
chr2 208976969 . C A 20118.23 PASS
AC=2;AF=1.00;AN=2;BaseQRankSum=-5.130e-01;ClippingRankSum=0.00;DP=22;ExcessHet=2.3742;FS=3.517;InbreedingCoeff=-0.0001;MQ=54.23;MQRankSum=0.00;QD=29.03;ReadPosRankSum=0.069;SOR=1.053;VQSLOD=2.72;culprit=MQRankSum GT:AD:DP:GQ:PGT:PID:PL
1/1:0,22:22:66:1|1:208976969_C_A:990,66,0
chr2 208976972 . G C 20932.62 PASS
AC=2;AF=1.00;AN=2;BaseQRankSum=1.70;ClippingRankSum=0.00;DP=23;ExcessHet=2.0222;FS=3.407;InbreedingCoeff=0.0062;MQ=53.83;MQRankSum=0.215;NEGATIVE_TRAIN_SITE;QD=26.84;ReadPosRankSum=1.18;SOR=1.059;VQSLOD=-2.082e+00;culprit=MQRankSum GT:AD:DP:GQ:PGT:PID:PL
1/1:0,23:23:75:1|1:208976969_C_A:1097,75,0
chr2 208976975 . T C 20935.23 PASS
AC=2;AF=1.00;AN=2;BaseQRankSum=2.29;ClippingRankSum=0.00;DP=25;ExcessHet=2.3742;FS=3.407;InbreedingCoeff=-0.0001;MQ=53.73;MQRankSum=0.215;NEGATIVE_TRAIN_SITE;QD=26.47;ReadPosRankSum=1.22;SOR=1.066;VQSLOD=-2.079e+00;culprit=MQRankSum
GT:AD:DP:GQ:PGT:PID:PL 1/1:0,25:25:75:1|1:208976969_C_A:1097,75,0
chr2 208977135 . A G 52181.63 PASS
AC=2;AF=1.00;AN=2;BaseQRankSum=-1.600e-01;ClippingRankSum=0.00;DP=133;ExcessHet=5.4449;FS=1.134;InbreedingCoeff=-0.1082;MQ=52.40;MQRankSum=-8.001e+00;QD=14.08;ReadPosRankSum=0.561;SOR=0.812;VQSLOD=-4.263e+00;culprit=MQRankSum GT:AD:DP:GQ:PL 1/1:0,133:133:99:3435,394,0
chr2 216262302 . CT C 1932.09 PASS
AC=2;AF=1.00;AN=2;BaseQRankSum=0.625;ClippingRankSum=0.00;DP=18;ExcessHet=0.5582;FS=10.474;InbreedingCoeff=0.2207;MQ=60.10;MQRankSum=0.00;POSITIVE_TRAIN_SITE;QD=18.23;ReadPosRankSum=1.27;SOR=3.308;VQSLOD=1.12;culprit=FS GT:AD:DP:GQ:PL 1/1:0,18:18:54:725,54,0
chr2 231326031 . G A 1091.25 PASS
AC=1;AF=0.500;AN=2;BaseQRankSum=1.01;ClippingRankSum=0.00;DP=45;ExcessHet=3.0605;FS=0.652;InbreedingCoeff=-0.0233;MQ=60.00;MQRankSum=0.00;POSITIVE_TRAIN_SITE;QD=8.20;ReadPosRankSum=0.393;SOR=0.607;VQSLOD=15.20;culprit=MQRankSum GT:AD:DP:GQ:PL
0/1:25,20:45:99:444,0,557
chr2 231380153 . C G 2013.25 PASS
AC=1;AF=0.500;AN=2;BaseQRankSum=0.845;ClippingRankSum=0.00;DP=95;ExcessHet=3.0605;FS=5.600;InbreedingCoeff=-0.0233;MQ=60.00;MQRankSum=0.00;QD=10.71;ReadPosRankSum=0.159;SOR=0.409;VQSLOD=13.96;culprit=MQRankSumGT:AD:DP:GQ:PL 0/1:50,45:95:99:1176,0,1297
chr2 231407704 . C T 528.28 PASS
AC=1;AF=0.500;AN=2;BaseQRankSum=-4.420e-01;ClippingRankSum=0.00;DP=20;ExcessHet=3.0605;FS=0.000;InbreedingCoeff=-0.0241;MQ=60.00;MQRankSum=0.00;POSITIVE_TRAIN_SITE;QD=16.01;ReadPosRankSum=1.17;SOR=0.437;VQSLOD=14.62;culprit=MQRankSum GT:AD:DP:GQ:PL
0/1:5,15:20:89:363,0,89
chr2 232790053 . C T 7794.89 PASS
AC=2;AF=1.00;AN=2;BaseQRankSum=0.079;ClippingRankSum=0.00;DP=10;ExcessHet=6.5616;FS=0.000;InbreedingCoeff=-0.1273;MQ=59.95;MQRankSum=0.00;POSITIVE_TRAIN_SITE;QD=20.14;ReadPosRankSu

```
m=-4.790e-01;SOR=0.732;VQSLOD=9.96;culprit=MQRankSum	GT:AD:DP:GQ:PL
	1/1:0,10:10:30:282,30,0
chr2	232952143	.	CTT	CT,CTTT,C	4283.73	PASS
	AC=2,0,0;AF=1.00,0.00,0.00;AN=2;BaseQRankSum=0.560;ClippingRankSum=
0.00;DP=16;ExcessHet=18.8691;FS=4.224;InbreedingCoeff=-
0.2275;MQ=63.16;MQRankSum=0.00;QD=6.78;ReadPosRankSum=0.674;SOR=1.036;VQS
LOD=5.13;culprit=MQRankSum	GT:AD:DP:GQ:PL
	1/1:0,16,0,0:16:48:406,48,0,406,48,406,406,48,406,406
chr2	239247174	.	A	G	11836.94	PASS
	AC=2;AF=1.00;AN=2;BaseQRankSum=-7.140e-
01;ClippingRankSum=0.00;DP=11;ExcessHet=3.2804;FS=0.565;InbreedingCoeff=-
0.0335;MQ=60.00;MQRankSum=0.00;POSITIVE_TRAIN_SITE;QD=15.95;ReadPosRankSu
m=-3.350e-01;SOR=0.612;VQSLOD=14.91;culprit=MQRankSum	GT:AD:DP:GQ:PL
	1/1:0,11:11:33:306,33,0
chr3	11596402	.	C	T	2659	PASS
	AC=2;AF=1.00;AN=2;BaseQRankSum=-6.380e-
01;ClippingRankSum=0.00;DP=19;ExcessHet=2.1168;FS=2.195;InbreedingCoeff=0
.0383;MQ=59.30;MQRankSum=0.00;POSITIVE_TRAIN_SITE;QD=10.59;ReadPosRankSum
=0.434;SOR=0.558;VQSLOD=7.48;culprit=MQRankSum	GT:AD:DP:GQ:PL
	1/1:0,19:19:57:595,57,0
chr3	53700304	.	G	A	10388.10	PASS
	AC=2;AF=1.00;AN=2;BaseQRankSum=1.22;ClippingRankSum=0.00;DP=102;Exc
essHet=1.6165;FS=0.000;InbreedingCoeff=0.0649;MQ=60.00;MQRankSum=0.00;POS
ITIVE_TRAIN_SITE;QD=12.64;ReadPosRankSum=-2.040e-
01;SOR=0.737;VQSLOD=15.18;culprit=MQRankSum	GT:AD:DP:GQ:PL
	1/1:1,101:102:99:2834,271,0
chr3	78706519	.	C	T	6616.46	PASS
	AC=2;AF=1.00;AN=2;BaseQRankSum=-
1.410e+00;ClippingRankSum=0.00;DP=20;ExcessHet=1.3801;FS=1.555;Inbreeding
Coeff=0.0593;MQ=59.97;MQRankSum=0.00;POSITIVE_TRAIN_SITE;QD=14.61;ReadPos
RankSum=0.531;SOR=0.847;VQSLOD=9.41;culprit=MQRankSum	GT:AD:DP:GQ:PL
	1/1:0,20:20:60:625,60,0
chr3	130135588	.	A	AT	37.97	PASS
	AC=1;AF=0.500;AN=2;BaseQRankSum=-7.230e-
01;ClippingRankSum=0.00;DP=16;ExcessHet=3.1627;FS=0.000;InbreedingCoeff=-
0.0427;MQ=129.83;MQRankSum=0.00;QD=0.97;ReadPosRankSum=0.137;SOR=0.752;VQ
SLOD=4.79;culprit=QD	GT:AD:DP:GQ:PL	0/1:13,3:16:38:38,0,293
chr3	178976821	.	G	A	16349.03	PASS
	AC=2;AF=1.00;AN=2;BaseQRankSum=1.10;ClippingRankSum=0.00;DP=26;Exce
ssHet=0.3703;FS=3.122;InbreedingCoeff=0.1880;MQ=58.80;MQRankSum=0.00;QD=2
0.18;ReadPosRankSum=-4.650e-01;SOR=0.466;VQSLOD=5.94;culprit=MQRankSum
	GT:AD:DP:GQ:PL	1/1:0,26:26:77:703,77,0
chr4	17503544	.	C	T	8835.14	PASS
	AC=2;AF=1.00;AN=2;BaseQRankSum=-
2.047e+00;ClippingRankSum=0.00;DP=20;ExcessHet=6.0814;FS=2.133;Inbreeding
Coeff=-
0.1229;MQ=60.00;MQRankSum=0.00;POSITIVE_TRAIN_SITE;QD=15.03;ReadPosRankSu
m=0.348;SOR=0.524;VQSLOD=14.79;culprit=MQRankSum	GT:AD:DP:GQ:PL
	1/1:1,19:20:50:555,50,0
chr4	38119885	.	G	A	21868.56	PASS
	AC=2;AF=1.00;AN=2;BaseQRankSum=-9.000e-
02;ClippingRankSum=0.00;DP=58;ExcessHet=5.7891;FS=3.537;InbreedingCoeff=-
0.1126;MQ=60.00;MQRankSum=0.00;POSITIVE_TRAIN_SITE;QD=12.95;ReadPosRankSu
m=-2.600e-01;SOR=0.951;VQSLOD=15.48;culprit=MQRankSum	GT:AD:DP:GQ:PL
	1/1:0,58:58:99:1778,174,0
chr4	39229771	.	G	A	9208.84	PASS
	AC=2;AF=1.00;AN=2;BaseQRankSum=2.03;ClippingRankSum=0.00;DP=33;Exce
ssHet=2.7005;FS=2.208;InbreedingCoeff=-
```

0.0007;MQ=60.00;MQRankSum=0.00;POSITIVE_TRAIN_SITE;QD=14.76;ReadPosRankSum=-2.760e-01;SOR=0.970;VQSLOD=15.65;culprit=MQRankSum GT:AD:DP:GQ:PL 1/1:0,33:33:99:1038,99,0
chr4 68780253 . G A 10819.55 PASS AC=2;AF=1.00;AN=2;BaseQRankSum=-1.408e+00;ClippingRankSum=0.00;DP=15;ExcessHet=4.1859;FS=1.226;InbreedingCoeff=-0.0998;MQ=59.98;MQRankSum=0.00;POSITIVE_TRAIN_SITE;QD=16.91;ReadPosRankSum=-2.960e-01;SOR=0.559;VQSLOD=9.73;culprit=MQRankSum GT:AD:DP:GQ:PL 1/1:0,15:15:45:429,45,0
chr4 68780512 . C A 6932.06 PASS AC=2;AF=1.00;AN=2;BaseQRankSum=-1.950e-01;ClippingRankSum=0.00;DP=12;ExcessHet=3.9515;FS=5.183;InbreedingCoeff=-0.0605;MQ=60.00;MQRankSum=0.00;POSITIVE_TRAIN_SITE;QD=15.00;ReadPosRankSum=0.092;SOR=1.090;VQSLOD=15.02;culprit=MQRankSum GT:AD:DP:GQ:PL 1/1:0,12:12:36:343,36,0
chr4 99802395 . C T 7029.62 PASS AC=2;AF=1.00;AN=2;BaseQRankSum=-1.744e+00;ClippingRankSum=0.00;DP=12;ExcessHet=2.6779;FS=4.015;InbreedingCoeff=0.0045;MQ=60.04;MQRankSum=0.00;POSITIVE_TRAIN_SITE;QD=15.22;ReadPosRankSum=-2.410e-01;SOR=0.392;VQSLOD=7.73;culprit=MQRankSum GT:AD:DP:GQ:PL 1/1:0,12:12:36:371,36,0
chr4 100265957 . C A 1940.84 PASS AC=2;AF=1.00;AN=2;BaseQRankSum=-1.732e+00;ClippingRankSum=0.00;DP=25;ExcessHet=0.7602;FS=0.691;InbreedingCoeff=0.1784;MQ=59.93;MQRankSum=0.00;POSITIVE_TRAIN_SITE;QD=14.93;ReadPosRankSum=-8.700e-01;SOR=0.818;VQSLOD=8.88;culprit=MQRankSum GT:AD:DP:GQ:PL 1/1:0,25:25:75:759,75,0
chr4 102992517 . T C 6417.36 PASS AC=2;AF=1.00;AN=2;BaseQRankSum=1.36;ClippingRankSum=0.00;DP=31;ExcessHet=0.5756;FS=0.558;InbreedingCoeff=0.1877;MQ=60.00;MQRankSum=0.00;POSITIVE_TRAIN_SITE;QD=15.81;ReadPosRankSum=-9.390e-01;SOR=0.617;VQSLOD=12.86;culprit=MQRankSum GT:AD:DP:GQ:PL 1/1:0,31:31:92:900,92,0
chr4 190874142 . G A 71.89 PASS AC=1;AF=0.500;AN=2;BaseQRankSum=-7.520e-01;ClippingRankSum=0.00;DP=26;ExcessHet=3.1702;FS=28.541;InbreedingCoeff=-0.0438;MQ=112.53;MQRankSum=-2.076e+00;QD=1.18;ReadPosRankSum=0.902;SOR=4.383;VQSLOD=-1.551e+01;culprit=MQ GT:AD:DP:GQ:PGT:PID:PL 0/1:23,3:26:45:0|1:190874142_G_A:45,0,1089
chr5 33988535 . C T 15516.49 PASS AC=2;AF=1.00;AN=2;BaseQRankSum=1.19;ClippingRankSum=0.00;DP=29;ExcessHet=0.1006;FS=0.000;InbreedingCoeff=0.3019;MQ=60.00;MQRankSum=0.00;POSITIVE_TRAIN_SITE;QD=17.55;ReadPosRankSum=0.238;SOR=0.693;VQSLOD=11.14;culprit=MQRankSum GT:AD:DP:GQ:PL 1/1:0,29:29:85:755,85,0
chr5 75923141 . G A 17296.56 PASS AC=2;AF=1.00;AN=2;BaseQRankSum=-1.996e+00;ClippingRankSum=0.00;DP=11;ExcessHet=2.1346;FS=3.487;InbreedingCoeff=0.0329;MQ=60.00;MQRankSum=0.00;POSITIVE_TRAIN_SITE;QD=18.90;ReadPosRankSum=0.165;SOR=1.009;VQSLOD=14.09;culprit=MQRankSum GT:AD:DP:GQ:PL 1/1:0,11:11:33:376,33,0
chr5 75927938 . A T 13691.48 PASS AC=2;AF=1.00;AN=2;BaseQRankSum=0.687;ClippingRankSum=0.00;DP=11;ExcessHet=3.1712;FS=0.000;InbreedingCoeff=-0.0341;MQ=60.00;MQRankSum=0.00;POSITIVE_TRAIN_SITE;QD=18.13;ReadPosRankSum=-3.000e-03;SOR=0.735;VQSLOD=14.91;culprit=MQRankSum GT:AD:DP:GQ:PL 1/1:0,11:11:33:374,33,0

```
chr5	75948474	.	T	C	14300.20	PASS
	AC=2;AF=1.00;AN=2;BaseQRankSum=1.72;ClippingRankSum=0.00;DP=16;ExcessHet=3.5264;FS=5.286;InbreedingCoeff=-0.0389;MQ=59.98;MQRankSum=0.00;POSITIVE_TRAIN_SITE;QD=19.43;ReadPosRankSum=0.270;SOR=1.267;VQSLOD=9.13;culprit=MQRankSum	GT:AD:DP:GQ:PL	1/1:0,16:16:48:530,48,0
chr5	89954184	.	T	C	24791.08	PASS
	AC=2;AF=1.00;AN=2;BaseQRankSum=-1.378e+00;ClippingRankSum=0.00;DP=44;ExcessHet=1.4405;FS=0.000;InbreedingCoeff=0.0782;MQ=59.96;MQRankSum=0.00;POSITIVE_TRAIN_SITE;QD=16.49;ReadPosRankSum=-2.420e-01;SOR=0.719;VQSLOD=10.84;culprit=MQRankSum	GT:AD:DP:GQ:PL	1/1:0,44:44:99:1184,130,0
chr5	89993045	.	T	C	5832.77	PASS
	AC=2;AF=1.00;AN=2;BaseQRankSum=2.10;ClippingRankSum=0.00;DP=10;ExcessHet=0.6047;FS=0.000;InbreedingCoeff=0.1454;MQ=60.00;MQRankSum=0.00;POSITIVE_TRAIN_SITE;QD=21.68;ReadPosRankSum=0.00;SOR=1.645;VQSLOD=11.26;culprit=MQRankSum	GT:AD:DP:GQ:PL	1/1:0,10:10:30:372,30,0
chr5	118529595	.	AATG	A	68.63	PASS
	AC=1;AF=0.500;AN=2;BaseQRankSum=-9.550e-01;ClippingRankSum=0.00;DP=36;ExcessHet=3.0103;FS=0.000;InbreedingCoeff=-0.0144;MQ=60.00;MQRankSum=0.00;QD=1.91;ReadPosRankSum=0.435;SOR=0.368;VQSLOD=4.75;culprit=QD	GT:AD:DP:GQ:PL	0/1:31,5:36:99:117,0,1333
chr5	155771421	.	A	AT	14960.88	PASS
	AC=2;AF=1.00;AN=2;BaseQRankSum=-3.200e-01;ClippingRankSum=0.00;DP=33;ExcessHet=5.4021;FS=1.799;InbreedingCoeff=-0.1015;MQ=60.00;MQRankSum=0.00;QD=12.27;ReadPosRankSum=-5.800e-02;SOR=0.848;VQSLOD=5.50;culprit=MQRankSum	GT:AD:DP:GQ:PL	1/1:0,33:33:99:937,99,0
chr5	156469783	.	G	A	5465.23	PASS
	AC=2;AF=1.00;AN=2;BaseQRankSum=-1.069e+00;ClippingRankSum=0.00;DP=18;ExcessHet=1.0591;FS=3.766;InbreedingCoeff=0.1149;MQ=60.00;MQRankSum=0.00;POSITIVE_TRAIN_SITE;QD=16.07;ReadPosRankSum=0.377;SOR=0.903;VQSLOD=14.62;culprit=MQRankSum	GT:AD:DP:GQ:PL	1/1:0,18:18:54:608,54,0
chr6	56334594	.	A	T	5529.24	PASS
	AC=2;AF=1.00;AN=2;BaseQRankSum=-1.399e+00;ClippingRankSum=0.00;DP=30;ExcessHet=1.0591;FS=1.281;InbreedingCoeff=0.1146;MQ=60.00;MQRankSum=0.00;POSITIVE_TRAIN_SITE;QD=14.71;ReadPosRankSum=-3.700e-02;SOR=0.613;VQSLOD=14.79;culprit=MQRankSum	GT:AD:DP:GQ:PL	1/1:0,30:30:90:923,90,0
chr6	70950375	.	G	GA	34.80	PASS
	AC=1;AF=0.500;AN=2;BaseQRankSum=-8.200e-02;ClippingRankSum=0.00;DP=38;ExcessHet=4.0535;FS=0.948;InbreedingCoeff=-0.0723;MQ=99.65;MQRankSum=0.00;QD=0.21;ReadPosRankSum=0.156;SOR=0.574;VQSLOD=4.77;culprit=QD	GT:AD:DP:GQ:PL	0/1:32,6:38:54:54,0,690
chr6	76550839	.	G	GT	22338.79	PASS
	AC=2;AF=1.00;AN=2;BaseQRankSum=-4.060e-01;ClippingRankSum=0.00;DP=14;ExcessHet=2.9987;FS=3.265;InbreedingCoeff=-0.0152;MQ=60.00;MQRankSum=0.00;POSITIVE_TRAIN_SITE;QD=23.13;ReadPosRankSum=-1.030e-01;SOR=0.433;VQSLOD=7.07;culprit=MQRankSum	GT:AD:DP:GQ:PL	1/1:0,14:14:42:560,42,0
chr6	79713405	.	T	TA	106.34	PASS
	AC=1;AF=0.500;AN=2;BaseQRankSum=-2.980e-01;ClippingRankSum=0.00;DP=16;ExcessHet=6.5830;FS=1.297;InbreedingCoeff=-0.1617;MQ=72.94;MQRankSum=0.00;QD=0.53;ReadPosRankSum=0.699;SOR=0.557;VQSLOD=4.78;culprit=QD	GT:AD:DP:GQ:PL	0/1:13,3:16:37:37,0,290
chr6	112441479	.	A	G	1156.25	PASS
	AC=1;AF=0.500;AN=2;BaseQRankSum=-5.460e-
```

01;ClippingRankSum=0.00;DP=61;ExcessHet=3.0605;FS=0.754;InbreedingCoeff=-0.0233;MQ=60.00;MQRankSum=0.00;QD=9.97;ReadPosRankSum=0.596;SOR=0.602;VQSLOD=14.15;culprit=MQRankSum GT:AD:DP:GQ:PL 0/1:31,30:61:99:635,0,684
chr6 112460365 . C T 2754.79 PASS
AC=1;AF=0.500;AN=2;BaseQRankSum=0.547;ClippingRankSum=0.00;DP=50;ExcessHet=3.8005;FS=0.427;InbreedingCoeff=-0.0732;MQ=59.91;MQRankSum=0.00;POSITIVE_TRAIN_SITE;QD=9.34;ReadPosRankSum=0.569;SOR=0.728;VQSLOD=7.60;culprit=MQRankSum GT:AD:DP:GQ:PL 0/1:25,25:50:99:533,0,514
chr6 128294709 . T A 4799.33 PASS
AC=2;AF=1.00;AN=2;BaseQRankSum=1.37;ClippingRankSum=0.00;DP=22;ExcessHet=1.1837;FS=2.477;InbreedingCoeff=0.1038;MQ=60.00;MQRankSum=0.00;POSITIVE_TRAIN_SITE;QD=16.05;ReadPosRankSum=0.00;SOR=0.475;VQSLOD=14.19;culprit=MQRankSum GT:AD:DP:GQ:PL 1/1:0,22:22:66:746,66,0
chr6 133782375 . T A 1344.16 PASS
AC=2;AF=1.00;AN=2;BaseQRankSum=-1.979e+00;ClippingRankSum=0.00;DP=12;ExcessHet=1.0275;FS=3.003;InbreedingCoeff=0.0995;MQ=60.00;MQRankSum=0.00;POSITIVE_TRAIN_SITE;QD=13.44;ReadPosRankSum=0.502;SOR=0.340;VQSLOD=14.96;culprit=MQRankSum GT:AD:DP:GQ:PL 1/1:0,12:12:36:362,36,0
chr6 147655367 . G GT 10.21 PASS
AC=1;AF=0.500;AN=2;BaseQRankSum=0.312;ClippingRankSum=0.00;DP=38;ExcessHet=3.0594;FS=1.501;InbreedingCoeff=-0.0175;MQ=76.33;MQRankSum=2.38;NEGATIVE_TRAIN_SITE;QD=0.27;ReadPosRankSum=-2.000e-02;SOR=1.158;VQSLOD=-3.010e+00;culprit=QD GT:AD:DP:GQ:PL 0/1:32,6:38:58:58,0,718
chr6 151293277 . C A 5118.64 PASS
AC=2;AF=1.00;AN=2;BaseQRankSum=-1.828e+00;ClippingRankSum=0.00;DP=20;ExcessHet=2.2393;FS=7.868;InbreedingCoeff=0.0191;MQ=60.00;MQRankSum=0.00;POSITIVE_TRAIN_SITE;QD=12.70;ReadPosRankSum=-5.020e-01;SOR=1.241;VQSLOD=13.27;culprit=MQRankSum GT:AD:DP:GQ:PL 1/1:0,20:20:60:584,60,0
chr6 160465753 . T G 26993.59 PASS
AC=2;AF=1.00;AN=2;BaseQRankSum=1.58;ClippingRankSum=0.00;DP=39;ExcessHet=7.2851;FS=3.548;InbreedingCoeff=-0.1513;MQ=59.97;MQRankSum=0.00;POSITIVE_TRAIN_SITE;QD=19.66;ReadPosRankSum=-1.380e-01;SOR=1.270;VQSLOD=8.42;culprit=MQRankSum GT:AD:DP:GQ:PL 1/1:0,39:39:99:1311,117,0
chr7 24324808 . C G 19426.64 PASS
AC=2;AF=1.00;AN=2;BaseQRankSum=1.03;ClippingRankSum=0.00;DP=62;ExcessHet=1.3050;FS=0.576;InbreedingCoeff=0.0754;MQ=59.98;MQRankSum=0.00;POSITIVE_TRAIN_SITE;QD=15.69;ReadPosRankSum=-2.230e-01;SOR=0.764;VQSLOD=10.65;culprit=MQRankSum GT:AD:DP:GQ:PL 1/1:0,62:62:99:1868,185,0
chr7 36656147 . A T 16696.64 PASS
AC=2;AF=1.00;AN=2;BaseQRankSum=0.470;ClippingRankSum=0.00;DP=28;ExcessHet=0.8601;FS=2.001;InbreedingCoeff=0.1146;MQ=60.00;MQRankSum=0.00;POSITIVE_TRAIN_SITE;QD=17.34;ReadPosRankSum=0.202;SOR=0.548;VQSLOD=14.36;culprit=MQRankSum GT:AD:DP:GQ:PL 1/1:0,28:28:84:844,84,0
chr7 37889786 . A G 42932.49 PASS
AC=2;AF=1.00;AN=2;BaseQRankSum=2.41;ClippingRankSum=0.00;DP=92;ExcessHet=2.8069;FS=4.039;InbreedingCoeff=-0.0021;MQ=60.00;MQRankSum=0.00;POSITIVE_TRAIN_SITE;QD=18.15;ReadPosRankSum=-5.620e-01;SOR=1.011;VQSLOD=14.37;culprit=MQRankSum GT:AD:DP:GQ:PL 1/1:0,92:92:99:2922,274,0
chr7 37903853 . A T 38344.33 PASS
AC=2;AF=1.00;AN=2;BaseQRankSum=-8.000e-02;ClippingRankSum=0.00;DP=50;ExcessHet=1.0168;FS=0.000;InbreedingCoeff=-

0.4410;MQ=60.00;MQRankSum=0.00;POSITIVE_TRAIN_SITE;QD=20.16;ReadPosRankSu
m=-3.940e-01;SOR=0.658;VQSLOD=10.44;culprit=MQRankSum GT:AD:DP:GQ:PL
1/1:0,50:50:99:1506,150,0
chr7 37904099 . G A 53701.29 PASS
AC=2;AF=1.00;AN=2;BaseQRankSum=0.240;ClippingRankSum=0.00;DP=51;Exc
essHet=1.0168;FS=0.000;InbreedingCoeff=0.1161;MQ=60.00;MQRankSum=0.00;POS
ITIVE_TRAIN_SITE;QD=19.70;ReadPosRankSum=-
1.010e+00;SOR=0.648;VQSLOD=13.07;culprit=MQRankSum GT:AD:DP:GQ:PL
1/1:0,51:51:99:1442,152,0
chr7 39491349 . C A 17.72 PASS
AC=1;AF=0.500;AN=2;BaseQRankSum=-
2.312e+00;ClippingRankSum=0.00;DP=14;ExcessHet=3.0103;FS=2.836;Inbreeding
Coeff=-0.0122;MQ=60.00;MQRankSum=0.00;QD=1.27;ReadPosRankSum=-4.950e-
01;SOR=0.105;VQSLOD=11.51;culprit=MQRankSum GT:AD:DP:GQ:PL
0/1:10,4:14:57:57,0,299
chr7 78119221 . G GAA,GA,GAAA 18227.14 PASS
AC=2,0,0;AF=1.00,0.00,0.00;AN=2;BaseQRankSum=0.230;ClippingRankSum=
0.00;DP=14;ExcessHet=3.0103;FS=0.000;InbreedingCoeff=0.2422;MQ=59.79;MQRa
nkSum=0.00;POSITIVE_TRAIN_SITE;QD=29.64;ReadPosRankSum=-1.160e-
01;SOR=0.934;VQSLOD=7.17;culprit=MQRankSum GT:AD:DP:GQ:PL
1/1:0,14,0,0:14:42:515,42,0,515,42,515,515,42,515,515
chr7 96635420 . AGCCGCCGCC AGCCGCC,AGCCGCCGCCGCC,A 28000.54
PASS
AC=1,0,0;AF=0.500,0.00,0.00;AN=2;BaseQRankSum=0.098;ClippingRankSum
=0.00;DP=131;ExcessHet=12.4563;FS=2.848;InbreedingCoeff=-
0.2753;MQ=59.82;MQRankSum=0.534;QD=13.57;ReadPosRankSum=-9.900e-
02;SOR=0.942;VQSLOD=-8.387e-01;culprit=MQRankSum GT:AD:DP:GQ:PL
0/1:119,12,0,0:131:99:147,0,4732,504,4768,5273,504,4768,5273,5273
chr7 97488350 . A G 4042.32 PASS
AC=2;AF=1.00;AN=2;BaseQRankSum=0.555;ClippingRankSum=0.00;DP=20;Exc
essHet=0.3496;FS=0.509;InbreedingCoeff=0.2076;MQ=59.90;MQRankSum=0.00;POS
ITIVE_TRAIN_SITE;QD=14.08;ReadPosRankSum=0.534;SOR=0.750;VQSLOD=7.09;culp
rit=MQRankSum GT:AD:DP:GQ:PL 1/1:0,20:20:60:626,60,0
chr7 100345106 . G A 6480.69 PASS
AC=2;AF=1.00;AN=2;BaseQRankSum=-
1.699e+00;ClippingRankSum=0.00;DP=30;ExcessHet=4.9489;FS=8.493;Inbreeding
Coeff=-
0.5994;MQ=60.00;MQRankSum=0.00;POSITIVE_TRAIN_SITE;QD=14.06;ReadPosRankSu
m=-1.260e-01;SOR=0.319;VQSLOD=8.85;culprit=MQRankSum GT:AD:DP:GQ:PL
1/1:0,30:30:90:919,90,0
chr7 100389928 . G T 4762.90 PASS
AC=2;AF=1.00;AN=2;BaseQRankSum=-
1.383e+00;ClippingRankSum=0.00;DP=10;ExcessHet=1.3042;FS=2.053;Inbreeding
Coeff=0.0072;MQ=59.80;MQRankSum=0.00;POSITIVE_TRAIN_SITE;QD=17.77;ReadPos
RankSum=0.852;SOR=0.587;VQSLOD=7.13;culprit=MQRankSum
GT:AD:DP:GQ:PGT:PID:PL 1/1:0,10:10:30:1|1:100389928_G_T:445,30,0
chr7 100389929 . A T 4762.03 PASS
AC=2;AF=1.00;AN=2;BaseQRankSum=0.792;ClippingRankSum=0.00;DP=10;Exc
essHet=1.3042;FS=2.053;InbreedingCoeff=0.0054;MQ=59.58;MQRankSum=0.00;POS
ITIVE_TRAIN_SITE;QD=17.77;ReadPosRankSum=0.852;SOR=0.587;VQSLOD=6.95;culp
rit=MQRankSum GT:AD:DP:GQ:PGT:PID:PL
1/1:0,10:10:30:1|1:100389928_G_T:445,30,0
chr7 134143690 . T C 11438.61 PASS
AC=2;AF=1.00;AN=2;BaseQRankSum=0.820;ClippingRankSum=0.00;DP=39;Exc
essHet=4.1735;FS=4.787;InbreedingCoeff=-
0.0702;MQ=60.00;MQRankSum=0.00;POSITIVE_TRAIN_SITE;QD=16.87;ReadPosRankSu
m=0.00;SOR=0.410;VQSLOD=15.07;culprit=MQRankSum GT:AD:DP:GQ:PL
1/1:0,39:39:99:1093,117,0

chr8 11702542 . T C 10035.50 PASS
AC=2;AF=1.00;AN=2;BaseQRankSum=2.19;ClippingRankSum=0.00;DP=10;ExcessHet=0.2120;FS=15.605;InbreedingCoeff=0.2334;MQ=59.68;MQRankSum=0.00;POSITIVE_TRAIN_SITE;QD=22.10;ReadPosRankSum=0.00;SOR=0.155;VQSLOD=8.35;culprit=MQRankSum GT:AD:DP:GQ:PL 1/1:0,10:10:30:323,30,0
chr8 17541784 . C T 5123.10 PASS
AC=2;AF=1.00;AN=2;BaseQRankSum=1.96;ClippingRankSum=0.00;DP=22;ExcessHet=2.9438;FS=13.684;InbreedingCoeff=-0.0193;MQ=59.94;MQRankSum=0.00;POSITIVE_TRAIN_SITE;QD=14.35;ReadPosRankSum=0.043;SOR=1.331;VQSLOD=8.07;culprit=MQRankSum GT:AD:DP:GQ:PL
1/1:0,22:22:66:750,66,0
chr8 22885154 . G A 42165.40 PASS
AC=2;AF=1.00;AN=2;BaseQRankSum=-1.236e+00;ClippingRankSum=0.00;DP=22;ExcessHet=6.3899;FS=0.588;InbreedingCoeff=-0.1282;MQ=60.00;MQRankSum=0.00;POSITIVE_TRAIN_SITE;QD=23.17;ReadPosRankSum=0.765;SOR=0.637;VQSLOD=11.65;culprit=MQRankSum GT:AD:DP:GQ:PGT:PID:PL
1/1:0,22:22:66:1|1:22885153_G_A:857,66,0
chr8 70512729 . A G 2729.42 PASS
AC=2;AF=1.00;AN=2;BaseQRankSum=1.65;ClippingRankSum=0.00;DP=14;ExcessHet=1.3247;FS=3.090;InbreedingCoeff=0.0468;MQ=60.00;MQRankSum=0.00;POSITIVE_TRAIN_SITE;QD=11.57;ReadPosRankSum=0.00;SOR=0.913;VQSLOD=15.60;culprit=MQRankSum GT:AD:DP:GQ:PL 1/1:0,14:14:42:499,42,0
chr8 129113373 . C T 35290.62 PASS
AC=2;AF=1.00;AN=2;BaseQRankSum=-3.150e-01;ClippingRankSum=0.00;DP=59;ExcessHet=0.1864;FS=38.850;InbreedingCoeff=0.2586;MQ=59.99;MQRankSum=0.00;POSITIVE_TRAIN_SITE;QD=21.68;ReadPosRankSum=0.483;SOR=0.015;VQSLOD=2.31;culprit=FS GT:AD:DP:GQ:PL
1/1:0,59:59:99:1661,175,0
chr8 133008823 . G A 31939.63 PASS
AC=2;AF=1.00;AN=2;BaseQRankSum=0.686;ClippingRankSum=0.00;DP=33;ExcessHet=4.1591;FS=0.692;InbreedingCoeff=-0.0698;MQ=59.96;MQRankSum=0.00;POSITIVE_TRAIN_SITE;QD=23.33;ReadPosRankSum=0.341;SOR=0.598;VQSLOD=10.67;culprit=MQRankSum GT:AD:DP:GQ:PL
1/1:0,33:33:98:965,98,0
chr8 133913525 . A G 5543.06 PASS
AC=2;AF=1.00;AN=2;BaseQRankSum=1.75;ClippingRankSum=0.00;DP=13;ExcessHet=0.0844;FS=3.568;InbreedingCoeff=0.3092;MQ=59.50;MQRankSum=0.00;POSITIVE_TRAIN_SITE;QD=18.60;ReadPosRankSum=0.501;SOR=1.009;VQSLOD=7.98;culprit=MQRankSum GT:AD:DP:GQ:PL 1/1:0,13:13:39:446,39,0
chr8 133984818 . TTTG T 27.51 PASS
AC=1;AF=0.500;AN=2;BaseQRankSum=-1.036e+00;ClippingRankSum=0.00;DP=5;ExcessHet=3.3048;FS=0.000;InbreedingCoeff=-0.0334;MQ=60.00;MQRankSum=0.00;QD=5.50;ReadPosRankSum=-6.320e-01;SOR=0.260;VQSLOD=5.01;culprit=QD GT:AD:DP:GQ:PGT:PID:PL
0/1:3,2:5:75:0|1:133984818_TTTG_T:75,0,120
chr9 734679 . G T 27845.13 PASS
AC=2;AF=1.00;AN=2;BaseQRankSum=-2.037e+00;ClippingRankSum=0.00;DP=38;ExcessHet=1.3843;FS=3.062;InbreedingCoeff=0.0806;MQ=60.01;MQRankSum=0.00;POSITIVE_TRAIN_SITE;QD=17.37;ReadPosRankSum=0.153;SOR=0.977;VQSLOD=8.34;culprit=MQRankSum GT:AD:DP:GQ:PL
1/1:0,38:38:99:1150,113,0
chr9 2182062 . A G 26700.86 PASS
AC=2;AF=1.00;AN=2;BaseQRankSum=-2.202e+00;ClippingRankSum=0.00;DP=39;ExcessHet=1.7878;FS=5.121;InbreedingCoeff=0.0539;MQ=59.99;MQRankSum=0.00;POSITIVE_TRAIN_SITE;QD=19.53;ReadPosRankSum=-1.620e-01;SOR=0.300;VQSLOD=9.69;culprit=MQRankSum
GT:AD:DP:GQ:PL 1/1:0,39:39:99:1086,117,0

chr9 6880020 . T C 379.26 PASS
AC=1;AF=0.500;AN=2;BaseQRankSum=0.302;ClippingRankSum=0.00;DP=23;ExcessHet=3.0605;FS=5.764;InbreedingCoeff=-0.0233;MQ=60.00;MQRankSum=0.00;QD=7.90;ReadPosRankSum=1.34;SOR=1.061;VQSLOD=13.35;culprit=MQRankSum GT:AD:DP:GQ:PL 0/1:12,11:23:99:203,0,216
chr9 7174813 . T A 3110.77 PASS
AC=1;AF=0.500;AN=2;BaseQRankSum=-1.049e+00;ClippingRankSum=0.00;DP=16;ExcessHet=2.4407;FS=14.805;InbreedingCoeff=0.0035;MQ=60.00;MQRankSum=0.00;POSITIVE_TRAIN_SITE;QD=11.52;ReadPosRankSum=-6.620e-01;SOR=0.290;VQSLOD=9.06;culprit=MQRankSum
GT:AD:DP:GQ:PL 0/1:10,6:16:99:110,0,250
chr9 74331376 . A C 19590.74 PASS
AC=2;AF=1.00;AN=2;BaseQRankSum=0.545;ClippingRankSum=0.00;DP=16;ExcessHet=4.5627;FS=0.000;InbreedingCoeff=-0.0773;MQ=60.00;MQRankSum=0.00;POSITIVE_TRAIN_SITE;QD=17.76;ReadPosRankSum=-2.230e-01;SOR=0.687;VQSLOD=14.85;culprit=MQRankSum GT:AD:DP:GQ:PL
1/1:0,16:16:48:483,48,0
chr9 77397519 . T C 13590.13 PASS
AC=2;AF=1.00;AN=2;BaseQRankSum=1.08;ClippingRankSum=0.00;DP=20;ExcessHet=20.4881;FS=0.524;InbreedingCoeff=-0.3757;MQ=60.00;MQRankSum=0.00;POSITIVE_TRAIN_SITE;QD=13.74;ReadPosRankSum=-4.520e-01;SOR=0.635;VQSLOD=10.51;culprit=MQRankSum
GT:AD:DP:GQ:PGT:PID:PL 1/1:0,20:20:60:1|1:77397519_T_C:692,60,0
chr9 79814799 . A G 51724.08 PASS
AC=2;AF=1.00;AN=2;BaseQRankSum=-2.642e+00;ClippingRankSum=0.00;DP=47;ExcessHet=1.2005;FS=1.384;InbreedingCoeff=0.0983;MQ=60.00;MQRankSum=0.00;POSITIVE_TRAIN_SITE;QD=18.92;ReadPosRankSum=-5.060e-01;SOR=0.856;VQSLOD=14.16;culprit=MQRankSum
GT:AD:DP:GQ:PL 1/1:0,47:47:99:1355,141,0
chr9 90537892 . G C 18687.68 PASS
AC=1;AF=0.500;AN=2;BaseQRankSum=1.62;ClippingRankSum=0.00;DP=43;ExcessHet=0.6959;FS=17.043;InbreedingCoeff=0.1994;MQ=43.14;MQRankSum=-4.261e+00;POSITIVE_TRAIN_SITE;QD=11.12;ReadPosRankSum=-3.310e-01;SOR=1.229;VQSLOD=-2.451e+00;culprit=MQRankSum GT:AD:DP:GQ:PL
0/1:34,9:43:99:157,0,931
chr10 5960209 . C T 36503.95 PASS
AC=2;AF=1.00;AN=2;BaseQRankSum=0.074;ClippingRankSum=0.00;DP=59;ExcessHet=0.8601;FS=1.343;InbreedingCoeff=0.1323;MQ=60.00;MQRankSum=0.00;POSITIVE_TRAIN_SITE;QD=19.56;ReadPosRankSum=-2.200e-02;SOR=0.803;VQSLOD=13.59;culprit=MQRankSum GT:AD:DP:GQ:PL
1/1:0,59:59:99:1934,177,0
chr10 26356066 . C T 13149.38 PASS
AC=2;AF=1.00;AN=2;BaseQRankSum=1.09;ClippingRankSum=0.00;DP=25;ExcessHet=0.8285;FS=2.505;InbreedingCoeff=0.1353;MQ=59.98;MQRankSum=0.00;POSITIVE_TRAIN_SITE;QD=19.54;ReadPosRankSum=-4.310e-01;SOR=1.018;VQSLOD=7.50;culprit=MQRankSum GT:AD:DP:GQ:PL
1/1:0,25:25:75:801,75,0
chr10 27349455 . A C 600.64 PASS
AC=1;AF=0.500;AN=2;BaseQRankSum=2.84;ClippingRankSum=0.00;DP=62;ExcessHet=3.0103;FS=4.454;InbreedingCoeff=-0.0122;MQ=60.00;MQRankSum=0.00;QD=9.69;ReadPosRankSum=1.30;SOR=1.507;VQSLOD=12.75;culprit=MQRankSum GT:AD:DP:GQ:PL 0/1:36,26:62:99:640,0,869
chr10 27703234 . A G 11267.89 PASS
AC=2;AF=1.00;AN=2;BaseQRankSum=0.364;ClippingRankSum=0.00;DP=24;ExcessHet=0.3689;FS=1.979;InbreedingCoeff=0.1944;MQ=60.00;MQRankSum=0.00;POSITIVE_TRAIN_SITE;QD=16.19;ReadPosRankSum=-2.320e-01;SOR=0.883;VQSLOD=13.35;culprit=MQRankSum GT:AD:DP:GQ:PL
1/1:0,24:24:70:670,70,0

chr10 54530636 . A G 65040.83 PASS
AC=2;AF=1.00;AN=2;BaseQRankSum=-5.040e-
01;ClippingRankSum=0.00;DP=47;ExcessHet=0.1013;FS=0.000;InbreedingCoeff=0
.3005;MQ=59.99;MQRankSum=0.00;POSITIVE_TRAIN_SITE;QD=21.78;ReadPosRankSum
=-3.680e-01;SOR=0.590;VQSLOD=10.90;culprit=MQRankSum
GT:AD:DP:GQ:PGT:PID:PL 1/1:0,47:47:99:1|1:54530636_A_G:1434,141,0
chr10 60273155 . C A 17734.26 PASS
AC=2;AF=1.00;AN=2;BaseQRankSum=0.00;ClippingRankSum=0.00;DP=27;Exce
ssHet=0.6874;FS=6.825;InbreedingCoeff=0.1498;MQ=60.00;MQRankSum=0.00;POSI
TIVE_TRAIN_SITE;QD=17.27;ReadPosRankSum=-6.870e-
01;SOR=0.962;VQSLOD=12.72;culprit=MQRankSum GT:AD:DP:GQ:PL
1/1:0,27:27:81:782,81,0
chr10 93768096 . G C 11332.33 PASS
AC=2;AF=1.00;AN=2;BaseQRankSum=-
1.865e+00;ClippingRankSum=0.00;DP=13;ExcessHet=4.5627;FS=1.826;Inbreeding
Coeff=-
0.0793;MQ=60.00;MQRankSum=0.00;QD=15.67;ReadPosRankSum=0.274;SOR=1.006;VQ
SLOD=13.62;culprit=MQRankSumGT:AD:DP:GQ:PL 1/1:0,13:13:39:390,39,0
chr10 97110879 . C T 20423.47 PASS
AC=2;AF=1.00;AN=2;BaseQRankSum=-2.190e-
01;ClippingRankSum=0.00;DP=24;ExcessHet=1.2812;FS=5.608;InbreedingCoeff=0
.0877;MQ=60.00;MQRankSum=0.00;POSITIVE_TRAIN_SITE;QD=23.67;ReadPosRankSum
=-1.690e-01;SOR=0.244;VQSLOD=12.17;culprit=MQRankSum GT:AD:DP:GQ:PL
1/1:0,24:24:72:705,72,0
chr10 97146712 . A T 15429.76 PASS
AC=2;AF=1.00;AN=2;BaseQRankSum=-3.380e-
01;ClippingRankSum=0.00;DP=17;ExcessHet=5.4235;FS=0.000;InbreedingCoeff=-
0.0995;MQ=60.00;MQRankSum=0.00;POSITIVE_TRAIN_SITE;QD=15.62;ReadPosRankSu
m=0.00;SOR=0.654;VQSLOD=14.71;culprit=MQRankSum GT:AD:DP:GQ:PL
1/1:0,17:17:51:448,51,0
chr10 97154682 . A T 8720.07 PASS
AC=2;AF=1.00;AN=2;BaseQRankSum=0.014;ClippingRankSum=0.00;DP=18;Exc
essHet=7.6752;FS=2.490;InbreedingCoeff=-
0.1468;MQ=59.96;MQRankSum=0.00;POSITIVE_TRAIN_SITE;QD=14.83;ReadPosRankSu
m=-3.300e-01;SOR=0.796;VQSLOD=7.99;culprit=MQRankSum GT:AD:DP:GQ:PL
1/1:0,18:18:53:498,53,0
chr10 97154698 . G A 16022.94 PASS
AC=2;AF=1.00;AN=2;BaseQRankSum=-4.730e-
01;ClippingRankSum=0.00;DP=33;ExcessHet=7.6752;FS=2.599;InbreedingCoeff=-
0.1592;MQ=59.95;MQRankSum=0.00;POSITIVE_TRAIN_SITE;QD=16.14;ReadPosRankSu
m=-9.920e-01;SOR=0.525;VQSLOD=7.07;culprit=MQRankSum GT:AD:DP:GQ:PL
1/1:0,33:33:99:940,99,0
chr10 97158713 . G A 15909.69 PASS
AC=2;AF=1.00;AN=2;BaseQRankSum=-3.540e-
01;ClippingRankSum=0.00;DP=46;ExcessHet=9.6106;FS=0.547;InbreedingCoeff=-
0.2036;MQ=60.00;MQRankSum=0.00;POSITIVE_TRAIN_SITE;QD=12.77;ReadPosRankSu
m=1.61;SOR=0.609;VQSLOD=14.19;culprit=MQRankSum GT:AD:DP:GQ:PL
1/1:0,46:46:99:1329,138,0
chr10 101293318 . G A 10314.35 PASS
AC=2;AF=1.00;AN=2;BaseQRankSum=-
1.037e+00;ClippingRankSum=0.00;DP=23;ExcessHet=0.6626;FS=1.586;Inbreeding
Coeff=0.1472;MQ=59.98;MQRankSum=0.00;POSITIVE_TRAIN_SITE;QD=19.03;ReadPos
RankSum=0.381;SOR=0.544;VQSLOD=9.26;culprit=MQRankSum GT:AD:DP:GQ:PL
1/1:0,23:23:68:707,68,0
chr10 101293341 . T C 7739.33 PASS
AC=2;AF=1.00;AN=2;BaseQRankSum=1.67;ClippingRankSum=0.00;DP=13;Exce
ssHet=0.6626;FS=4.216;InbreedingCoeff=0.1368;MQ=59.97;MQRankSum=0.00;POSI
TIVE_TRAIN_SITE;QD=22.83;ReadPosRankSum=-1.610e-

01;SOR=1.447;VQSLOD=8.48;culprit=MQRankSum	GT:AD:DP:GQ:PL
	1/1:0,13:13:39:454,39,0
chr10	103565960	.	G	C	30191.45	PASS
	AC=2;AF=1.00;AN=2;BaseQRankSum=2.46;ClippingRankSum=0.00;DP=20;Exce
ssHet=0.6874;FS=3.596;InbreedingCoeff=0.1532;MQ=60.00;MQRankSum=0.00;POSI
TIVE_TRAIN_SITE;QD=21.32;ReadPosRankSum=-4.160e-
01;SOR=0.457;VQSLOD=12.75;culprit=MQRankSum	GT:AD:DP:GQ:PL
	1/1:0,20:20:60:693,60,0
chr10	114169190	.	C	A	18679.52	PASS
	AC=2;AF=1.00;AN=2;BaseQRankSum=0.293;ClippingRankSum=0.00;DP=26;Exc
essHet=0.9269;FS=1.347;InbreedingCoeff=0.1250;MQ=60.00;MQRankSum=0.00;POS
ITIVE_TRAIN_SITE;QD=18.77;ReadPosRankSum=0.482;SOR=0.827;VQSLOD=13.87;cul
prit=MQRankSum	GT:AD:DP:GQ:PL	1/1:0,26:26:77:771,77,0
chr11	694949	.	TGCCGCGGCCGCGGCCGCCGCCGCCACAGCG	T	62.83	PASS
	AC=1;AF=0.500;AN=2;BaseQRankSum=0.063;ClippingRankSum=0.00;DP=24;Ex
cessHet=3.0103;FS=0.000;InbreedingCoeff=-
0.0268;MQ=102.13;MQRankSum=3.31;QD=2.62;ReadPosRankSum=0.813;SOR=0.669;VQ
SLOD=-1.105e-01;culprit=MQRankSum	GT:AD:DP:GQ:PL
	0/1:20,4:24:99:110,0,1177
chr11	8474291	.	T	C	1532.39	PASS
	AC=2;AF=1.00;AN=2;BaseQRankSum=0.547;ClippingRankSum=0.00;DP=15;Exc
essHet=0.5722;FS=1.704;InbreedingCoeff=0.1969;MQ=60.00;MQRankSum=0.00;POS
ITIVE_TRAIN_SITE;QD=13.93;ReadPosRankSum=0.261;SOR=0.928;VQSLOD=12.97;cul
prit=MQRankSum	GT:AD:DP:GQ:PL	1/1:0,15:15:45:481,45,0
chr11	20959482	.	G	C	33499.34	PASS
	AC=2;AF=1.00;AN=2;BaseQRankSum=1.16;ClippingRankSum=0.00;DP=37;Exce
ssHet=2.2393;FS=0.000;InbreedingCoeff=0.0188;MQ=60.11;MQRankSum=0.00;POSI
TIVE_TRAIN_SITE;QD=26.01;ReadPosRankSum=0.122;SOR=0.726;VQSLOD=7.74;culpr
it=MQRankSum	GT:AD:DP:GQ:PL	1/1:0,37:37:99:1252,111,0
chr11	47644331	.	A	ACAGTTGTTGGCAGCCAT	262.56	PASS
	AC=1;AF=0.500;AN=2;BaseQRankSum=0.813;ClippingRankSum=0.00;DP=36;Ex
cessHet=3.3188;FS=40.417;InbreedingCoeff=-
0.0441;MQ=74.47;MQRankSum=0.00;NEGATIVE_TRAIN_SITE;QD=2.10;ReadPosRankSum
=-2.216e+00;SOR=4.467;VQSLOD=-4.420e+00;culprit=ReadPosRankSum
	GT:AD:DP:GQ:PGT:PID:PL
	0/1:32,4:36:71:0|1:47644331_A_ACAGTTGTTGGCAGCCAT:71,0,1788
chr11	47644333	.	A	AGATTGGAGACAAGCACAGAGGGATAGGTCAAC	262.57
	PASS
	AC=1;AF=0.500;AN=2;BaseQRankSum=2.71;ClippingRankSum=0.00;DP=36;Exc
essHet=3.3188;FS=34.236;InbreedingCoeff=-
0.4820;MQ=76.54;MQRankSum=0.00;NEGATIVE_TRAIN_SITE;QD=2.71;ReadPosRankSum
=-2.296e+00;SOR=4.270;VQSLOD=-3.803e+00;culprit=ReadPosRankSum
	GT:AD:DP:GQ:PGT:PID:PL
	0/1:32,4:36:71:0|1:47644331_A_ACAGTTGTTGGCAGCCAT:71,0,1788
chr11	59858036	.	C	G	4830.58	PASS
	AC=2;AF=1.00;AN=2;BaseQRankSum=-
1.368e+00;ClippingRankSum=0.00;DP=13;ExcessHet=1.6263;FS=26.295;Inbreedin
gCoeff=0.0342;MQ=59.85;MQRankSum=0.00;POSITIVE_TRAIN_SITE;QD=17.19;ReadPo
sRankSum=0.00;SOR=2.683;VQSLOD=5.34;culprit=MQRankSum	GT:AD:DP:GQ:PL
	1/1:0,13:13:39:468,39,0
chr11	73498933	.	T	G	7289.43	PASS
	AC=2;AF=1.00;AN=2;BaseQRankSum=-
2.157e+00;ClippingRankSum=0.00;DP=14;ExcessHet=1.5815;FS=0.578;Inbreeding
Coeff=0.0525;MQ=60.00;MQRankSum=0.00;POSITIVE_TRAIN_SITE;QD=15.61;ReadPos
RankSum=-3.660e-01;SOR=0.761;VQSLOD=15.29;culprit=MQRankSum
	GT:AD:DP:GQ:PL	1/1:0,14:14:41:387,41,0
chr11	75110680	.	C	T	2431.66	PASS
	AC=2;AF=1.00;AN=2;BaseQRankSum=0.510;ClippingRankSum=0.00;DP=20;Exc

essHet=0.4910;FS=5.466;InbreedingCoeff=0.2080;MQ=57.95;MQRankSum=0.00;POSITIVE_TRAIN_SITE;QD=14.56;ReadPosRankSum=-1.070e-01;SOR=1.152;VQSLOD=7.13;culprit=MQRankSum GT:AD:DP:GQ:PL 1/1:0,20:20:59:574,59,0
chr11 102586186 . AAC A 118.93 PASS AC=1;AF=0.500;AN=2;BaseQRankSum=-1.348e+00;ClippingRankSum=0.00;DP=32;ExcessHet=3.1627;FS=3.189;InbreedingCoeff=-0.0394;MQ=60.00;MQRankSum=0.00;POSITIVE_TRAIN_SITE;QD=1.75;ReadPosRankSum=2.66;SOR=0.255;VQSLOD=3.84;culprit=ReadPosRankSum GT:AD:DP:GQ:PL 0/1:26,6:32:99:134,0,896
chr11 124747333 . A G 13538.88 PASS AC=2;AF=1.00;AN=2;BaseQRankSum=1.56;ClippingRankSum=0.00;DP=37;ExcessHet=0.5540;FS=3.755;InbreedingCoeff=0.1429;MQ=58.68;MQRankSum=0.00;POSITIVE_TRAIN_SITE;QD=19.91;ReadPosRankSum=-6.050e-01;SOR=1.033;VQSLOD=7.85;culprit=MQRankSum GT:AD:DP:GQ:PL 1/1:0,37:37:99:1138,110,0
chr12 2055266 . T C 25463.25 PASS AC=2;AF=1.00;AN=2;BaseQRankSum=1.83;ClippingRankSum=0.00;DP=26;ExcessHet=4.5627;FS=1.936;InbreedingCoeff=-0.0733;MQ=59.98;MQRankSum=0.00;POSITIVE_TRAIN_SITE;QD=20.52;ReadPosRankSum=0.052;SOR=0.616;VQSLOD=9.66;culprit=MQRankSum GT:AD:DP:GQ:PL 1/1:0,26:26:78:869,78,0
chr12 2107036 . T C 37970.64 PASS AC=2;AF=1.00;AN=2;BaseQRankSum=-1.130e+00;ClippingRankSum=0.00;DP=64;ExcessHet=5.8072;FS=0.543;InbreedingCoeff=-0.1116;MQ=59.98;MQRankSum=0.00;POSITIVE_TRAIN_SITE;QD=14.48;ReadPosRankSum=-2.960e-01;SOR=0.631;VQSLOD=10.97;culprit=MQRankSum GT:AD:DP:GQ:PL 1/1:0,64:64:99:1801,191,0
chr12 21014178 . A G 8057.62 PASS AC=2;AF=1.00;AN=2;BaseQRankSum=0.933;ClippingRankSum=0.00;DP=16;ExcessHet=1.3852;FS=5.021;InbreedingCoeff=0.0828;MQ=60.00;MQRankSum=0.00;POSITIVE_TRAIN_SITE;QD=15.62;ReadPosRankSum=-4.460e-01;SOR=0.408;VQSLOD=14.97;culprit=MQRankSum GT:AD:DP:GQ:PL 1/1:0,16:16:48:554,48,0
chr12 21728874 . C T 3415.41 PASS AC=2;AF=1.00;AN=2;BaseQRankSum=1.69;ClippingRankSum=0.00;DP=79;ExcessHet=0.1524;FS=0.000;InbreedingCoeff=0.4762;MQ=60.00;MQRankSum=0.00;POSITIVE_TRAIN_SITE;QD=15.89;ReadPosRankSum=-3.920e-01;SOR=0.730;VQSLOD=9.81;culprit=MQRankSum GT:AD:DP:GQ:PL 1/1:0,79:79:99:2142,236,0
chr12 22047091 . C T 6075.41 PASS AC=2;AF=1.00;AN=2;BaseQRankSum=0.357;ClippingRankSum=0.00;DP=136;ExcessHet=0.1524;FS=0.000;InbreedingCoeff=0.4762;MQ=60.00;MQRankSum=0.00;POSITIVE_TRAIN_SITE;QD=14.64;ReadPosRankSum=0.921;SOR=0.656;VQSLOD=9.38;culprit=MQRankSum GT:AD:DP:GQ:PL 1/1:0,136:136:99:3588,406,0
chr12 32446835 . G A 8325.76 PASS AC=2;AF=1.00;AN=2;BaseQRankSum=-1.960e+00;ClippingRankSum=0.00;DP=13;ExcessHet=2.6493;FS=1.632;InbreedingCoeff=-0.0279;MQ=60.00;MQRankSum=0.00;POSITIVE_TRAIN_SITE;QD=18.79;ReadPosRankSum=0.115;SOR=0.584;VQSLOD=14.70;culprit=MQRankSum GT:AD:DP:GQ:PL 1/1:0,13:13:39:375,39,0
chr12 48375475 . C G 13360.41 PASS AC=2;AF=1.00;AN=2;BaseQRankSum=0.853;ClippingRankSum=0.00;DP=35;ExcessHet=6.3899;FS=2.541;InbreedingCoeff=-0.1282;MQ=60.00;MQRankSum=0.00;POSITIVE_TRAIN_SITE;QD=15.11;ReadPosRankSu

m=0.493;SOR=0.881;VQSLOD=15.06;culprit=MQRankSum GT:AD:DP:GQ:PL
1/1:0,35:35:99:1107,105,0
chr12 48376447 . A G 14553.28 PASS
AC=2;AF=1.00;AN=2;BaseQRankSum=-5.900e-01;ClippingRankSum=0.00;DP=14;ExcessHet=2.0002;FS=1.913;InbreedingCoeff=-0.0068;MQ=59.98;MQRankSum=0.00;QD=27.77;ReadPosRankSum=0.00;SOR=0.554;VQSLOD=7.28;culprit=MQRankSum GT:AD:DP:GQ:PGT:PID:PL
1/1:0,14:14:42:1|1:48376443_A_AC:630,42,0
chr12 48376530 . C A 10765.10 PASS
AC=2;AF=1.00;AN=2;BaseQRankSum=-1.050e-01;ClippingRankSum=0.00;DP=15;ExcessHet=9.5151;FS=1.209;InbreedingCoeff=-0.2123;MQ=59.95;MQRankSum=0.00;POSITIVE_TRAIN_SITE;QD=16.24;ReadPosRankSum=0.943;SOR=0.796;VQSLOD=9.51;culprit=MQRankSum GT:AD:DP:GQ:PL
1/1:0,15:15:45:494,45,0
chr12 48379810 . G A 10957.37 PASS
AC=2;AF=1.00;AN=2;BaseQRankSum=-7.690e-01;ClippingRankSum=0.00;DP=32;ExcessHet=1.4405;FS=1.186;InbreedingCoeff=0.0762;MQ=60.00;MQRankSum=0.00;POSITIVE_TRAIN_SITE;QD=16.07;ReadPosRankSum=0.00;SOR=0.568;VQSLOD=14.69;culprit=MQRankSum GT:AD:DP:GQ:PL
1/1:0,32:32:96:919,96,0
chr12 48379856 . T C 6454.28 PASS
AC=2;AF=1.00;AN=2;BaseQRankSum=1.47;ClippingRankSum=0.00;DP=10;ExcessHet=5.1295;FS=0.000;InbreedingCoeff=-0.1168;MQ=59.20;MQRankSum=0.00;POSITIVE_TRAIN_SITE;QD=19.10;ReadPosRankSum=0.253;SOR=0.702;VQSLOD=7.12;culprit=MQRankSum GT:AD:DP:GQ:PL
1/1:0,10:10:30:348,30,0
chr12 48380031 . A G 14325.46 PASS
AC=2;AF=1.00;AN=2;BaseQRankSum=1.37;ClippingRankSum=0.00;DP=18;ExcessHet=9.6106;FS=4.063;InbreedingCoeff=-0.2079;MQ=60.00;MQRankSum=0.00;POSITIVE_TRAIN_SITE;QD=20.09;ReadPosRankSum=-8.400e-02;SOR=0.442;VQSLOD=12.39;culprit=MQRankSum GT:AD:DP:GQ:PL
1/1:0,18:18:53:573,53,0
chr12 54575800 . C A 12817.49 PASS
AC=2;AF=1.00;AN=2;BaseQRankSum=-2.387e+00;ClippingRankSum=0.00;DP=17;ExcessHet=4.5301;FS=0.708;InbreedingCoeff=-0.0719;MQ=60.00;MQRankSum=0.00;POSITIVE_TRAIN_SITE;QD=16.52;ReadPosRankSum=-8.900e-02;SOR=0.580;VQSLOD=15.19;culprit=MQRankSum GT:AD:DP:GQ:PL
1/1:0,17:17:51:521,51,0
chr12 121711768 . T G 2462.52 PASS
AC=2;AF=1.00;AN=2;BaseQRankSum=-1.834e+00;ClippingRankSum=0.00;DP=10;ExcessHet=0.0005;FS=6.787;InbreedingCoeff=0.4546;MQ=59.73;MQRankSum=0.00;POSITIVE_TRAIN_SITE;QD=22.39;ReadPosRankSum=-5.660e-01;SOR=0.112;VQSLOD=8.09;culprit=MQRankSum
GT:AD:DP:GQ:PL 1/1:0,10:10:30:307,30,0
chr12 125453277 . G A 6290.37 PASS
AC=2;AF=1.00;AN=2;BaseQRankSum=-4.970e-01;ClippingRankSum=0.00;DP=13;ExcessHet=2.5460;FS=0.000;InbreedingCoeff=0.0108;MQ=60.00;MQRankSum=0.00;POSITIVE_TRAIN_SITE;QD=12.00;ReadPosRankSum=0.195;SOR=0.741;VQSLOD=15.56;culprit=MQRankSum GT:AD:DP:GQ:PL
1/1:0,13:13:39:399,39,0
chr12 125457203 . G A 7429.45 PASS
AC=2;AF=1.00;AN=2;BaseQRankSum=0.611;ClippingRankSum=0.00;DP=31;ExcessHet=2.1311;FS=3.449;InbreedingCoeff=0.0300;MQ=60.00;MQRankSum=0.00;POSITIVE_TRAIN_SITE;QD=12.24;ReadPosRankSum=-4.280e-01;SOR=0.954;VQSLOD=15.12;culprit=MQRankSum GT:AD:DP:GQ:PL
1/1:0,31:31:92:903,92,0

chr12 133435595 . C T 4065.60 PASS
AC=2;AF=1.00;AN=2;BaseQRankSum=-7.390e-
01;ClippingRankSum=0.00;DP=17;ExcessHet=5.5569;FS=2.886;InbreedingCoeff=-
0.1417;MQ=60.00;MQRankSum=0.00;POSITIVE_TRAIN_SITE;QD=13.83;ReadPosRankSu
m=0.079;SOR=0.933;VQSLOD=14.61;culprit=MQRankSum GT:AD:DP:GQ:PL
1/1:0,17:17:51:564,51,0
chr13 31843415 . G A 481.26 PASS
AC=1;AF=0.500;AN=2;BaseQRankSum=-2.030e-
01;ClippingRankSum=0.00;DP=20;ExcessHet=3.0605;FS=0.000;InbreedingCoeff=-
0.0236;MQ=60.00;MQRankSum=0.00;QD=15.04;ReadPosRankSum=1.05;SOR=0.906;VQS
LOD=13.80;culprit=MQRankSum GT:AD:DP:GQ:PL 0/1:6,14:20:97:310,0,97
chr13 31848739 . AC A 553.21 PASS
AC=1;AF=0.500;AN=2;BaseQRankSum=3.00;ClippingRankSum=0.00;DP=22;Exc
essHet=3.0605;FS=3.997;InbreedingCoeff=-
0.0233;MQ=60.00;MQRankSum=0.00;QD=10.85;ReadPosRankSum=1.49;SOR=1.292;VQS
LOD=4.86;culprit=MQRankSum GT:AD:DP:GQ:PL 0/1:19,3:22:55:55,0,670
chr13 50087142 . T C 5646.25 PASS
AC=2;AF=1.00;AN=2;BaseQRankSum=1.77;ClippingRankSum=0.00;DP=10;Exce
ssHet=2.5617;FS=9.404;InbreedingCoeff=-
0.0056;MQ=60.00;MQRankSum=0.00;POSITIVE_TRAIN_SITE;QD=14.26;ReadPosRankSu
m=0.431;SOR=1.159;VQSLOD=11.90;culprit=MQRankSum GT:AD:DP:GQ:PL
1/1:0,10:10:30:329,30,0
chr13 50096127 . C G 10383.01 PASS
AC=2;AF=1.00;AN=2;BaseQRankSum=0.332;ClippingRankSum=0.00;DP=15;Exc
essHet=3.0355;FS=3.385;InbreedingCoeff=-
0.0149;MQ=60.00;MQRankSum=0.00;POSITIVE_TRAIN_SITE;QD=14.60;ReadPosRankSu
m=-2.310e-01;SOR=0.941;VQSLOD=15.67;culprit=MQRankSum GT:AD:DP:GQ:PL
1/1:0,15:15:45:472,45,0
chr13 75873787 . G A 14064.75 PASS
AC=2;AF=1.00;AN=2;BaseQRankSum=-
1.915e+00;ClippingRankSum=0.00;DP=15;ExcessHet=9.0243;FS=7.720;Inbreeding
Coeff=-
0.1915;MQ=60.00;MQRankSum=0.00;POSITIVE_TRAIN_SITE;QD=19.59;ReadPosRankSu
m=-3.720e-01;SOR=0.103;VQSLOD=10.95;culprit=MQRankSum GT:AD:DP:GQ:PL
1/1:0,15:15:45:511,45,0
chr13 75880384 . CAA C,CA 38679.61 PASS
AC=2,0;AF=1.00,0.00;AN=2;BaseQRankSum=0.565;ClippingRankSum=0.00;DP
=15;ExcessHet=4.9806;FS=0.000;InbreedingCoeff=-
0.1061;MQ=59.99;MQRankSum=0.00;POSITIVE_TRAIN_SITE;QD=25.67;ReadPosRankSu
m=0.025;SOR=0.624;VQSLOD=7.18;culprit=MQRankSum GT:AD:DP:GQ:PL
1/1:0,13,0:15:45:501,45,0,462,47,449
chr13 75880667 . G A 35320.14 PASS
AC=2;AF=1.00;AN=2;BaseQRankSum=-2.300e-
02;ClippingRankSum=0.00;DP=38;ExcessHet=8.5822;FS=0.000;InbreedingCoeff=-
0.1818;MQ=59.97;MQRankSum=0.00;POSITIVE_TRAIN_SITE;QD=15.51;ReadPosRankSu
m=0.797;SOR=0.686;VQSLOD=10.56;culprit=MQRankSum GT:AD:DP:GQ:PL
1/1:0,38:38:99:1077,113,0
chr13 75923536 . T TA 21265.91 PASS
AC=2;AF=1.00;AN=2;BaseQRankSum=-3.820e-
01;ClippingRankSum=0.00;DP=24;ExcessHet=5.9473;FS=3.360;InbreedingCoeff=-
0.1138;MQ=60.00;MQRankSum=0.00;POSITIVE_TRAIN_SITE;QD=17.25;ReadPosRankSu
m=-7.450e-01;SOR=0.799;VQSLOD=6.72;culprit=MQRankSum GT:AD:DP:GQ:PL
1/1:0,24:24:72:758,72,0
chr13 111098110 . C T 27430.08 PASS
AC=2;AF=1.00;AN=2;BaseQRankSum=0.263;ClippingRankSum=0.00;DP=20;Exc
essHet=6.7712;FS=0.000;InbreedingCoeff=-
0.1580;MQ=60.00;MQRankSum=0.00;POSITIVE_TRAIN_SITE;QD=23.83;ReadPosRankSu

m=-1.091e+00;SOR=0.730;VQSLOD=11.36;culprit=MQRankSum GT:AD:DP:GQ:PL
1/1:0,20:20:59:603,59,0
chr13 111099057 . G A 32560.34 PASS
AC=2;AF=1.00;AN=2;BaseQRankSum=0.959;ClippingRankSum=0.00;DP=33;ExcessHet=4.8828;FS=0.000;InbreedingCoeff=-0.0936;MQ=60.00;MQRankSum=0.00;POSITIVE_TRAIN_SITE;QD=22.07;ReadPosRankSum=0.231;SOR=0.598;VQSLOD=12.66;culprit=MQRankSum GT:AD:DP:GQ:PL
1/1:0,33:33:99:974,99,0
chr14 23310732 . C T 1658.25 PASS
AC=1;AF=0.500;AN=2;BaseQRankSum=1.29;ClippingRankSum=0.00;DP=44;ExcessHet=3.0605;FS=0.771;InbreedingCoeff=-0.0233;MQ=60.00;MQRankSum=0.00;QD=14.67;ReadPosRankSum=2.05;SOR=0.852;VQSLOD=13.48;culprit=MQRankSum GT:AD:DP:GQ:PL 0/1:16,28:44:99:658,0,294
chr14 23313994 . G C 937.26 PASS
AC=1;AF=0.500;AN=2;BaseQRankSum=0.711;ClippingRankSum=0.00;DP=30;ExcessHet=3.0605;FS=0.000;InbreedingCoeff=-0.0236;MQ=60.00;MQRankSum=0.00;QD=13.99;ReadPosRankSum=-1.320e-01;SOR=0.793;VQSLOD=14.08;culprit=MQRankSum GT:AD:DP:GQ:PL
0/1:10,20:30:99:486,0,226
chr14 23882144 . T C 17462.46 PASS
AC=2;AF=1.00;AN=2;BaseQRankSum=-8.360e-01;ClippingRankSum=0.00;DP=65;ExcessHet=2.2926;FS=0.000;InbreedingCoeff=0.0228;MQ=60.00;MQRankSum=0.00;POSITIVE_TRAIN_SITE;QD=13.95;ReadPosRankSum=0.255;SOR=0.750;VQSLOD=15.68;culprit=MQRankSum GT:AD:DP:GQ:PL
1/1:0,65:65:99:1621,192,0
chr14 23882186 . T G 8186.27 PASS
AC=2;AF=1.00;AN=2;BaseQRankSum=-1.170e-01;ClippingRankSum=0.00;DP=27;ExcessHet=2.2926;FS=4.520;InbreedingCoeff=0.0413;MQ=60.00;MQRankSum=0.00;POSITIVE_TRAIN_SITE;QD=17.05;ReadPosRankSum=-3.140e-01;SOR=1.029;VQSLOD=15.02;culprit=MQRankSum GT:AD:DP:GQ:PL
1/1:0,27:27:81:797,81,0
chr14 23885997 . G A 3539.60 PASS
AC=2;AF=1.00;AN=2;BaseQRankSum=2.19;ClippingRankSum=0.00;DP=13;ExcessHet=0.7902;FS=3.844;InbreedingCoeff=0.0909;MQ=59.87;MQRankSum=0.00;POSITIVE_TRAIN_SITE;QD=15.87;ReadPosRankSum=0.00;SOR=1.107;VQSLOD=8.51;culprit=MQRankSum GT:AD:DP:GQ:PL 1/1:0,13:13:39:426,39,0
chr14 23888323 . T TG 14559.35 PASS
AC=2;AF=1.00;AN=2;BaseQRankSum=0.468;ClippingRankSum=0.00;DP=29;ExcessHet=7.2523;FS=0.000;InbreedingCoeff=-0.1628;MQ=60.00;MQRankSum=0.00;POSITIVE_TRAIN_SITE;QD=20.42;ReadPosRankSum=-3.660e-01;SOR=0.733;VQSLOD=7.05;culprit=MQRankSum
GT:AD:DP:GQ:PGT:PID:PL 1/1:0,29:29:87:1|1:23888323_T_TG:1119,87,0
chr14 74534274 . TA T 33.92 PASS
AC=1;AF=0.500;AN=2;BaseQRankSum=-8.700e-02;ClippingRankSum=0.00;DP=44;ExcessHet=3.7639;FS=0.000;InbreedingCoeff=-0.0617;MQ=117.62;MQRankSum=0.00;QD=0.27;ReadPosRankSum=-3.600e-02;SOR=0.752;VQSLOD=4.77;culprit=QD GT:AD:DP:GQ:PL
0/1:37,6:44:31:31,0,949
chr14 77895502 . T C 1420.70 PASS
AC=2;AF=1.00;AN=2;BaseQRankSum=2.83;ClippingRankSum=0.00;DP=32;ExcessHet=0.1524;FS=4.432;InbreedingCoeff=0.4142;MQ=60.00;MQRankSum=0.00;POSITIVE_TRAIN_SITE;QD=16.33;ReadPosRankSum=2.60;SOR=0.326;VQSLOD=7.96;culprit=MQRankSum GT:AD:DP:GQ:PL 1/1:0,32:32:94:932,94,0
chr14 78063823 . AT A 8068.07 PASS
AC=2;AF=1.00;AN=2;BaseQRankSum=-9.800e-02;ClippingRankSum=0.00;DP=10;ExcessHet=3.3098;FS=5.425;InbreedingCoeff=-0.0388;MQ=60.00;MQRankSum=0.00;POSITIVE_TRAIN_SITE;QD=17.43;ReadPosRankSu

m=-2.300e-01;SOR=1.316;VQSLOD=6.93;culprit=MQRankSum GT:AD:DP:GQ:PL 1/1:0,10:10:30:321,30,0
chr14 81422257 . G C 4733.95 PASS AC=2;AF=1.00;AN=2;BaseQRankSum=0.415;ClippingRankSum=0.00;DP=27;ExcessHet=2.4107;FS=1.028;InbreedingCoeff=-0.0151;MQ=59.98;MQRankSum=0.00;POSITIVE_TRAIN_SITE;QD=14.35;ReadPosRankSum=0.00;SOR=0.799;VQSLOD=10.87;culprit=MQRankSum GT:AD:DP:GQ:PL 1/1:0,27:27:81:802,81,0
chr14 89310261 . A G 2407.70 PASS AC=2;AF=1.00;AN=2;BaseQRankSum=0.574;ClippingRankSum=0.00;DP=26;ExcessHet=1.0012;FS=4.602;InbreedingCoeff=0.1293;MQ=60.00;MQRankSum=0.00;POSITIVE_TRAIN_SITE;QD=14.42;ReadPosRankSum=0.726;SOR=0.429;VQSLOD=14.53;culprit=MQRankSum GT:AD:DP:GQ:PL 1/1:0,26:26:78:807,78,0
chr15 25364795 . C T 40646.40 PASS AC=2;AF=1.00;AN=2;BaseQRankSum=-1.969e+00;ClippingRankSum=0.00;DP=90;ExcessHet=6.3899;FS=0.534;InbreedingCoeff=-0.1282;MQ=59.92;MQRankSum=0.00;POSITIVE_TRAIN_SITE;QD=13.95;ReadPosRankSum=-6.180e-01;SOR=0.625;VQSLOD=8.70;culprit=MQRankSum GT:AD:DP:GQ:PL 1/1:0,90:90:99:2618,269,0
chr15 28516084 . C T 6250.49 PASS AC=2;AF=1.00;AN=2;BaseQRankSum=-1.282e+00;ClippingRankSum=0.00;DP=10;ExcessHet=4.3244;FS=15.395;InbreedingCoeff=0.0694;MQ=58.57;MQRankSum=0.00;POSITIVE_TRAIN_SITE;QD=26.37;ReadPosRankSum=0.366;SOR=2.671;VQSLOD=6.27;culprit=MQRankSum GT:AD:DP:GQ:PL 1/1:0,10:10:30:310,30,0
chr15 34077949 . G A 5686.69 PASS AC=1;AF=0.500;AN=2;BaseQRankSum=-9.970e-01;ClippingRankSum=0.00;DP=167;ExcessHet=3.1627;FS=1.743;InbreedingCoeff=-0.0353;MQ=60.00;MQRankSum=0.00;QD=9.80;ReadPosRankSum=0.572;SOR=0.544;VQSLOD=14.12;culprit=MQRankSum GT:AD:DP:GQ:PL 0/1:91,76:167:99:1567,0,1996
chr15 34112906 . C A 22154.22 PASS AC=2;AF=1.00;AN=2;BaseQRankSum=-9.310e-01;ClippingRankSum=0.00;DP=19;ExcessHet=2.7005;FS=1.609;InbreedingCoeff=-0.0110;MQ=60.00;MQRankSum=0.00;POSITIVE_TRAIN_SITE;QD=21.08;ReadPosRankSum=0.176;SOR=0.525;VQSLOD=13.84;culprit=MQRankSum GT:AD:DP:GQ:PL 1/1:0,19:19:57:564,57,0
chr15 34150083 . G A 6195.43 PASS AC=1;AF=0.500;AN=2;BaseQRankSum=0.749;ClippingRankSum=0.00;DP=158;ExcessHet=3.3188;FS=0.518;InbreedingCoeff=-0.0476;MQ=59.98;MQRankSum=0.00;QD=10.59;ReadPosRankSum=0.604;SOR=0.745;VQSLOD=8.99;culprit=MQRankSum GT:AD:DP:GQ:PL 0/1:66,92:158:99:1954,0,1247
chr15 43694108 . G C 10692.06 PASS AC=2;AF=1.00;AN=2;BaseQRankSum=0.381;ClippingRankSum=0.00;DP=41;ExcessHet=0.1349;FS=1.225;InbreedingCoeff=0.3205;MQ=60.00;MQRankSum=0.00;POSITIVE_TRAIN_SITE;QD=18.63;ReadPosRankSum=0.165;SOR=0.771;VQSLOD=10.64;culprit=MQRankSum GT:AD:DP:GQ:PL 1/1:0,41:41:99:1252,121,0
chr15 50924995 . G A 33247.82 PASS AC=2;AF=1.00;AN=2;BaseQRankSum=-1.083e+00;ClippingRankSum=0.00;DP=25;ExcessHet=0.6874;FS=2.051;InbreedingCoeff=0.1593;MQ=59.99;MQRankSum=0.00;POSITIVE_TRAIN_SITE;QD=20.95;ReadPosRankSum=0.644;SOR=0.493;VQSLOD=8.69;culprit=MQRankSum GT:AD:DP:GQ:PL 1/1:0,25:25:75:776,75,0
chr15 50926523 . AAAAT A 40948.37 PASS AC=2;AF=1.00;AN=2;BaseQRankSum=0.262;ClippingRankSum=0.00;DP=32;ExcessHet=0.5205;FS=0.000;InbreedingCoeff=0.1759;MQ=59.99;MQRankSum=0.00;QD=

33.84;ReadPosRankSum=0.494;SOR=0.739;VQSLOD=5.76;culprit=MQRankSum
GT:AD:DP:GQ:PL 1/1:0,32:32:96:1440,96,0
chr15 86182512 . A G 6913.19 PASS
AC=2;AF=1.00;AN=2;BaseQRankSum=1.07;ClippingRankSum=0.00;DP=17;ExcessHet=9.3966;FS=0.527;InbreedingCoeff=-0.2048;MQ=60.00;MQRankSum=0.00;POSITIVE_TRAIN_SITE;QD=13.56;ReadPosRankSum=0.883;SOR=0.765;VQSLOD=13.78;culprit=MQRankSum GT:AD:DP:GQ:PL
1/1:0,17:17:51:586,51,0
chr15 86212866 . A G 5898.23 PASS
AC=2;AF=1.00;AN=2;BaseQRankSum=2.36;ClippingRankSum=0.00;DP=12;ExcessHet=6.4504;FS=19.234;InbreedingCoeff=-0.1438;MQ=59.95;MQRankSum=0.00;POSITIVE_TRAIN_SITE;QD=15.77;ReadPosRankSum=-6.510e-01;SOR=1.849;VQSLOD=6.92;culprit=MQRankSum GT:AD:DP:GQ:PL
1/1:0,12:12:36:421,36,0
chr15 88726586 . G A 5541.50 PASS
AC=2;AF=1.00;AN=2;BaseQRankSum=1.18;ClippingRankSum=0.00;DP=26;ExcessHet=1.6533;FS=0.396;InbreedingCoeff=0.0590;MQ=60.00;MQRankSum=0.00;POSITIVE_TRAIN_SITE;QD=15.65;ReadPosRankSum=0.658;SOR=0.683;VQSLOD=14.64;culprit=MQRankSum GT:AD:DP:GQ:PL 1/1:1,25:26:50:701,50,0
chr15 93567526 . G A 22521.87 PASS
AC=2;AF=1.00;AN=2;BaseQRankSum=-3.258e+00;ClippingRankSum=0.00;DP=36;ExcessHet=0.3703;FS=3.266;InbreedingCoeff=0.2071;MQ=60.00;MQRankSum=0.00;POSITIVE_TRAIN_SITE;QD=21.51;ReadPosRankSum=0.261;SOR=1.155;VQSLOD=11.14;culprit=MQRankSum GT:AD:DP:GQ:PL
1/1:0,36:36:99:1181,107,0
chr15 100636741 . C A 14.02 PASS
AC=1;AF=0.500;AN=2;BaseQRankSum=-2.638e+00;ClippingRankSum=0.00;DP=10;ExcessHet=3.0594;FS=5.229;InbreedingCoeff=-0.0183;MQ=60.00;MQRankSum=0.00;QD=1.40;ReadPosRankSum=-1.780e+00;SOR=0.035;VQSLOD=10.54;culprit=MQRankSum GT:AD:DP:GQ:PL
0/1:7,3:10:53:53,0,231
chr16 1261024 . C T 9340.49 PASS
AC=2;AF=1.00;AN=2;BaseQRankSum=1.82;ClippingRankSum=0.00;DP=12;ExcessHet=0.0516;FS=0.793;InbreedingCoeff=0.2762;MQ=59.93;MQRankSum=0.00;POSITIVE_TRAIN_SITE;QD=21.57;ReadPosRankSum=0.313;SOR=0.909;VQSLOD=8.07;culprit=MQRankSum GT:AD:DP:GQ:PL 1/1:0,12:12:36:414,36,0
chr16 1261377 . G A 17666.71 PASS
AC=2;AF=1.00;AN=2;BaseQRankSum=-1.383e+00;ClippingRankSum=0.00;DP=24;ExcessHet=0.7046;FS=0.000;InbreedingCoeff=0.1605;MQ=59.97;MQRankSum=0.00;POSITIVE_TRAIN_SITE;QD=21.03;ReadPosRankSum=0.431;SOR=0.667;VQSLOD=10.54;culprit=MQRankSum GT:AD:DP:GQ:PL
1/1:0,24:24:72:747,72,0
chr16 1560882 . T C 505.35 PASS
AC=1;AF=0.500;AN=2;BaseQRankSum=-1.887e+00;ClippingRankSum=0.00;DP=21;ExcessHet=3.0605;FS=5.379;InbreedingCoeff=-0.0266;MQ=60.00;MQRankSum=0.00;POSITIVE_TRAIN_SITE;QD=9.36;ReadPosRankSum=0.931;SOR=0.545;VQSLOD=15.07;culprit=MQRankSum GT:AD:DP:GQ:PL
0/1:9,12:21:99:251,0,208
chr16 1652418 . C T 3382.43 PASS
AC=1;AF=0.500;AN=2;BaseQRankSum=0.011;ClippingRankSum=0.00;DP=75;ExcessHet=3.3188;FS=1.846;InbreedingCoeff=-0.0476;MQ=60.00;MQRankSum=0.00;QD=10.07;ReadPosRankSum=0.695;SOR=0.811;VQSLOD=14.15;culprit=MQRankSumGT:AD:DP:GQ:PL 0/1:33,42:75:99:904,0,620
chr16 16286614 . G C 21127.59 PASS
AC=2;AF=1.00;AN=2;BaseQRankSum=1.20;ClippingRankSum=0.00;DP=33;ExcessHet=2.1655;FS=2.209;InbreedingCoeff=0.0213;MQ=60.00;MQRankSum=0.00;POSI

TIVE_TRAIN_SITE;QD=18.95;ReadPosRankSum=0.190;SOR=0.940;VQSLOD=14.12;culprit=MQRankSum GT:AD:DP:GQ:PL 1/1:0,33:33:99:1015,99,0
chr16 58051178 . C T 17421.95 PASS
AC=2;AF=1.00;AN=2;BaseQRankSum=-1.800e-02;ClippingRankSum=0.00;DP=26;ExcessHet=12.4465;FS=3.913;InbreedingCoeff=-0.2567;MQ=60.00;MQRankSum=0.00;POSITIVE_TRAIN_SITE;QD=18.30;ReadPosRankSum=-4.130e-01;SOR=1.002;VQSLOD=12.08;culprit=MQRankSum GT:AD:DP:GQ:PL
1/1:0,26:26:77:747,77,0
chr16 67974227 . G A 4665.25 PASS
AC=1;AF=0.500;AN=2;BaseQRankSum=0.263;ClippingRankSum=0.00;DP=233;ExcessHet=3.0605;FS=4.149;InbreedingCoeff=-0.0233;MQ=60.00;MQRankSum=0.00;QD=10.60;ReadPosRankSum=1.28;SOR=0.462;VQSLOD=13.92;culprit=MQRankSum GT:AD:DP:GQ:PL
0/1:101,132:233:99:2994,0,2157
chr16 67976824 . G A 1898.26 PASS
AC=1;AF=0.500;AN=2;BaseQRankSum=1.66;ClippingRankSum=0.00;DP=98;ExcessHet=3.0605;FS=3.349;InbreedingCoeff=-0.0233;MQ=60.00;MQRankSum=0.00;QD=10.10;ReadPosRankSum=1.28;SOR=0.966;VQSLOD=13.96;culprit=MQRankSum GT:AD:DP:GQ:PL 0/1:50,48:98:99:1093,0,1068
chr16 72048632 . A G 4134.88 PASS
AC=2;AF=1.00;AN=2;BaseQRankSum=0.691;ClippingRankSum=0.00;DP=11;ExcessHet=4.1510;FS=1.943;InbreedingCoeff=-0.0804;MQ=60.00;MQRankSum=0.00;POSITIVE_TRAIN_SITE;QD=13.47;ReadPosRankSum=-4.890e-01;SOR=0.886;VQSLOD=15.67;culprit=MQRankSum GT:AD:DP:GQ:PL
1/1:0,11:11:33:340,33,0
chr17 1635883 . C T 17589.57 PASS
AC=2;AF=1.00;AN=2;BaseQRankSum=0.069;ClippingRankSum=0.00;DP=55;ExcessHet=0.8627;FS=0.000;InbreedingCoeff=0.1445;MQ=60.00;MQRankSum=0.00;POSITIVE_TRAIN_SITE;QD=15.86;ReadPosRankSum=0.116;SOR=0.653;VQSLOD=14.21;culprit=MQRankSum GT:AD:DP:GQ:PL 1/1:0,55:55:99:1619,163,0
chr17 8158273 . A G 16217.69 PASS
AC=2;AF=1.00;AN=2;BaseQRankSum=1.61;ClippingRankSum=0.00;DP=18;ExcessHet=4.8828;FS=0.000;InbreedingCoeff=-0.1223;MQ=60.00;MQRankSum=0.00;POSITIVE_TRAIN_SITE;QD=23.30;ReadPosRankSum=-7.900e-02;SOR=0.783;VQSLOD=12.15;culprit=MQRankSum GT:AD:DP:GQ:PL
1/1:0,18:18:53:548,53,0
chr17 11666674 . T C 22686.35 PASS
AC=2;AF=1.00;AN=2;BaseQRankSum=-1.250e-01;ClippingRankSum=0.00;DP=26;ExcessHet=4.7648;FS=4.111;InbreedingCoeff=-0.0805;MQ=60.00;MQRankSum=0.00;POSITIVE_TRAIN_SITE;QD=20.04;ReadPosRankSum=0.296;SOR=0.411;VQSLOD=14.00;culprit=MQRankSum GT:AD:DP:GQ:PL
1/1:0,26:26:77:713,77,0
chr17 12896102 . C A 426.44 PASS
AC=1;AF=0.500;AN=2;BaseQRankSum=1.67;ClippingRankSum=0.00;DP=9;ExcessHet=3.0686;FS=0.000;InbreedingCoeff=-0.0530;MQ=60.00;MQRankSum=0.00;QD=17.06;ReadPosRankSum=0.431;SOR=1.358;VQSLOD=13.17;culprit=MQRankSumGT:AD:DP:GQ:PL 0/1:6,3:9:78:78,0,127
chr17 12921348 . G C 2728.93 PASS
AC=1;AF=0.500;AN=2;BaseQRankSum=0.282;ClippingRankSum=0.00;DP=29;ExcessHet=4.7383;FS=0.429;InbreedingCoeff=-0.1202;MQ=60.00;MQRankSum=0.00;POSITIVE_TRAIN_SITE;QD=8.72;ReadPosRankSum=0.728;SOR=0.658;VQSLOD=15.00;culprit=MQRankSum GT:AD:DP:GQ:PGT:PID:PL
0/1:19,10:29:99:0|1:12921348_G_C:198,0,476
chr17 38066240 . T C 6646.80 PASS
AC=2;AF=1.00;AN=2;BaseQRankSum=1.83;ClippingRankSum=0.00;DP=11;ExcessHet=1.6376;FS=0.000;InbreedingCoeff=0.0609;MQ=60.00;MQRankSum=0.00;POSITIVE_TRAIN_SITE;QD=20.51;ReadPosRankSum=-3.930e-

01;SOR=0.796;VQSLOD=13.91;culprit=MQRankSum GT:AD:DP:GQ:PL
1/1:0,11:11:33:357,33,0
chr17 40724276 . C T 4957.27 PASS
AC=2;AF=1.00;AN=2;BaseQRankSum=0.605;ClippingRankSum=0.00;DP=12;ExcessHet=1.4531;FS=0.721;InbreedingCoeff=0.0379;MQ=60.00;MQRankSum=0.00;POSITIVE_TRAIN_SITE;QD=17.27;ReadPosRankSum=0.00;SOR=0.847;VQSLOD=15.05;culprit=MQRankSum GT:AD:DP:GQ:PL 1/1:0,12:12:36:397,36,0
chr17 67152893 . CAAAT C 42553.89 PASS
AC=2;AF=1.00;AN=2;BaseQRankSum=-4.140e-01;ClippingRankSum=0.00;DP=18;ExcessHet=0.2201;FS=0.000;InbreedingCoeff=0.2574;MQ=59.98;MQRankSum=0.00;POSITIVE_TRAIN_SITE;QD=27.90;ReadPosRankSum=0.085;SOR=0.742;VQSLOD=7.14;culprit=MQRankSum GT:AD:DP:GQ:PL
1/1:0,18:18:54:810,54,0
chr17 67181548 . T G 26767.93 PASS
AC=2;AF=1.00;AN=2;BaseQRankSum=-1.111e+00;ClippingRankSum=0.00;DP=14;ExcessHet=0.3118;FS=3.119;InbreedingCoeff=0.2253;MQ=59.96;MQRankSum=0.00;POSITIVE_TRAIN_SITE;QD=29.29;ReadPosRankSum=0.686;SOR=0.946;VQSLOD=7.21;culprit=MQRankSum
GT:AD:DP:GQ:PGT:PID:PL 1/1:0,14:14:42:1|1:67181540_C_A:496,42,0
chr17 67211058 . T A 27556.84 PASS
AC=2;AF=1.00;AN=2;BaseQRankSum=0.963;ClippingRankSum=0.00;DP=10;ExcessHet=0.3118;FS=1.441;InbreedingCoeff=0.2262;MQ=59.98;MQRankSum=0.00;POSITIVE_TRAIN_SITE;QD=22.59;ReadPosRankSum=0.066;SOR=0.907;VQSLOD=9.16;culprit=MQRankSum GT:AD:DP:GQ:PL 1/1:0,10:10:30:327,30,0
chr17 79767614 . CCTGT C 128.35 PASS
AC=1;AF=0.500;AN=2;BaseQRankSum=1.56;ClippingRankSum=0.00;DP=23;ExcessHet=3.0605;FS=1.329;InbreedingCoeff=-0.0272;MQ=88.09;MQRankSum=-7.350e-01;NEGATIVE_TRAIN_SITE;QD=2.85;ReadPosRankSum=0.471;SOR=0.997;VQSLOD=-1.841e+00;culprit=MQRankSum GT:AD:DP:GQ:PL 0/1:18,5:23:99:121,0,784
chr18 3131593 . CT C,CTT 4624.70 PASS
AC=2,0;AF=1.00,0.00;AN=2;BaseQRankSum=0.103;ClippingRankSum=0.00;DP=10;ExcessHet=14.8412;FS=0.000;InbreedingCoeff=-0.2465;MQ=63.56;MQRankSum=0.00;POSITIVE_TRAIN_SITE;QD=7.89;ReadPosRankSum=0.075;SOR=0.676;VQSLOD=6.76;culprit=MQRankSum GT:AD:DP:GQ:PL
1/1:0,10,0:10:30:261,30,0,261,30,261
chr18 3173873 . G A 17634.58 PASS
AC=2;AF=1.00;AN=2;BaseQRankSum=-1.491e+00;ClippingRankSum=0.00;DP=45;ExcessHet=0.9243;FS=0.000;InbreedingCoeff=0.1266;MQ=60.00;MQRankSum=0.00;POSITIVE_TRAIN_SITE;QD=14.59;ReadPosRankSum=-1.670e-01;SOR=0.702;VQSLOD=14.65;culprit=MQRankSum
GT:AD:DP:GQ:PL 1/1:0,45:45:99:1193,134,0
chr18 3174056 . C A 35795.58 PASS
AC=2;AF=1.00;AN=2;BaseQRankSum=-1.330e+00;ClippingRankSum=0.00;DP=44;ExcessHet=0.9243;FS=0.000;InbreedingCoeff=0.1266;MQ=60.01;MQRankSum=0.00;POSITIVE_TRAIN_SITE;QD=22.63;ReadPosRankSum=-9.640e-01;SOR=0.703;VQSLOD=10.15;culprit=MQRankSum
GT:AD:DP:GQ:PGT:PID:PL 1/1:0,44:44:99:1|1:3174056_C_A:1969,132,0
chr18 3174064 . G T 32694.84 PASS
AC=2;AF=1.00;AN=2;BaseQRankSum=-1.878e+00;ClippingRankSum=0.00;DP=44;ExcessHet=2.7005;FS=0.000;InbreedingCoeff=-0.0007;MQ=60.00;MQRankSum=0.00;QD=23.07;ReadPosRankSum=-9.740e-01;SOR=0.716;VQSLOD=11.18;culprit=MQRankSum GT:AD:DP:GQ:PGT:PID:PL
1/1:0,44:44:99:1|1:3174056_C_A:2007,135,0
chr18 47113008 . C T 10542.98 PASS
AC=2;AF=1.00;AN=2;BaseQRankSum=-1.356e+00;ClippingRankSum=0.00;DP=16;ExcessHet=0.2985;FS=1.260;InbreedingCoeff=0.2040;MQ=60.27;MQRankSum=0.00;POSITIVE_TRAIN_SITE;QD=19.03;ReadPos

RankSum=-2.100e-01;SOR=0.813;VQSLOD=7.35;culprit=MQRankSum
GT:AD:DP:GQ:PL 1/1:0,16:16:48:486,48,0
chr19 617205 . T C 16407.72 PASS
AC=2;AF=1.00;AN=2;BaseQRankSum=0.780;ClippingRankSum=0.00;DP=27;ExcessHet=1.7556;FS=1.310;InbreedingCoeff=0.0576;MQ=59.97;MQRankSum=0.00;POSITIVE_TRAIN_SITE;QD=18.41;ReadPosRankSum=-8.880e-01;SOR=0.832;VQSLOD=9.69;culprit=MQRankSum GT:AD:DP:GQ:PL
1/1:0,27:27:81:833,81,0
chr19 13356150 . T G 19726.83 PASS
AC=2;AF=1.00;AN=2;BaseQRankSum=1.10;ClippingRankSum=0.00;DP=29;ExcessHet=14.7586;FS=1.245;InbreedingCoeff=-0.2976;MQ=60.00;MQRankSum=0.00;POSITIVE_TRAIN_SITE;QD=17.43;ReadPosRankSum=-2.820e-01;SOR=0.578;VQSLOD=11.44;culprit=MQRankSum GT:AD:DP:GQ:PL
1/1:0,29:29:87:887,87,0
chr19 38948356 . T C 16075.86 PASS
AC=2;AF=1.00;AN=2;BaseQRankSum=2.28;ClippingRankSum=0.00;DP=27;ExcessHet=1.3050;FS=7.536;InbreedingCoeff=0.0868;MQ=60.00;MQRankSum=0.00;POSITIVE_TRAIN_SITE;QD=25.40;ReadPosRankSum=-5.490e-01;SOR=0.143;VQSLOD=10.37;culprit=MQRankSum GT:AD:DP:GQ:PL
1/1:0,27:27:81:1004,81,0
chr19 38949729 . T G 35744.60 PASS
AC=2;AF=1.00;AN=2;BaseQRankSum=1.70;ClippingRankSum=0.00;DP=21;ExcessHet=2.9748;FS=0.739;InbreedingCoeff=-0.0118;MQ=60.00;MQRankSum=0.00;POSITIVE_TRAIN_SITE;QD=22.57;ReadPosRankSum=-4.120e-01;SOR=0.550;VQSLOD=13.18;culprit=MQRankSum GT:AD:DP:GQ:PL
1/1:0,21:21:62:714,62,0
chr19 38950947 . C T 12057.97 PASS
AC=2;AF=1.00;AN=2;BaseQRankSum=-5.620e-01;ClippingRankSum=0.00;DP=13;ExcessHet=1.1606;FS=4.391;InbreedingCoeff=0.1100;MQ=60.00;MQRankSum=0.00;POSITIVE_TRAIN_SITE;QD=22.41;ReadPosRankSum=0.023;SOR=0.385;VQSLOD=12.81;culprit=MQRankSum GT:AD:DP:GQ:PL
1/1:0,13:13:39:455,39,0
chr19 41107416 . C T 1038.91 PASS
AC=1;AF=0.500;AN=2;BaseQRankSum=1.99;ClippingRankSum=0.00;DP=57;ExcessHet=3.0686;FS=0.000;InbreedingCoeff=-0.0365;MQ=60.00;MQRankSum=0.00;QD=9.99;ReadPosRankSum=1.50;SOR=0.758;VQSLOD=14.03;culprit=MQRankSum GT:AD:DP:GQ:PL 0/1:27,30:57:99:604,0,551
chr19 41107428 . C T 3359.31 PASS
AC=1;AF=0.500;AN=2;BaseQRankSum=0.112;ClippingRankSum=0.00;DP=53;ExcessHet=3.9097;FS=0.834;InbreedingCoeff=-0.0989;MQ=60.00;MQRankSum=0.00;QD=10.00;ReadPosRankSum=-3.560e-01;SOR=0.767;VQSLOD=14.02;culprit=MQRankSum GT:AD:DP:GQ:PL
0/1:29,24:53:99:505,0,567
chr19 41518773 . C T 5083.57 PASS
AC=2;AF=1.00;AN=2;BaseQRankSum=-1.123e+00;ClippingRankSum=0.00;DP=10;ExcessHet=6.0814;FS=0.456;InbreedingCoeff=-0.1267;MQ=59.16;MQRankSum=-6.230e-01;POSITIVE_TRAIN_SITE;QD=13.10;ReadPosRankSum=0.00;SOR=0.655;VQSLOD=1.66;culprit=MQRankSum GT:AD:DP:GQ:PL 1/1:0,10:10:30:302,30,0
chr19 47109011 . T C 35076.41 PASS
AC=2;AF=1.00;AN=2;BaseQRankSum=0.734;ClippingRankSum=0.00;DP=69;ExcessHet=0.1710;FS=0.000;InbreedingCoeff=0.2737;MQ=60.00;MQRankSum=0.00;POSITIVE_TRAIN_SITE;QD=22.51;ReadPosRankSum=-3.090e-01;SOR=0.694;VQSLOD=10.70;culprit=MQRankSum GT:AD:DP:GQ:PL
1/1:0,69:69:99:1989,206,0
chr19 47111663 . T C 32220.57 PASS
AC=2;AF=1.00;AN=2;BaseQRankSum=0.144;ClippingRankSum=0.00;DP=34;ExcessHet=0.2762;FS=1.575;InbreedingCoeff=0.2358;MQ=59.98;MQRankSum=0.00;POS

```
ITIVE_TRAIN_SITE;QD=23.62;ReadPosRankSum=0.654;SOR=0.550;VQSLOD=9.18;culp
rit=MQRankSum    GT:AD:DP:GQ:PL   1/1:0,34:34:99:1112,102,0
chr19 48182522    .     A     G     10544.55    PASS
      AC=2;AF=1.00;AN=2;BaseQRankSum=1.66;ClippingRankSum=0.00;DP=28;Exce
ssHet=6.9227;FS=1.189;InbreedingCoeff=-
0.1458;MQ=59.97;MQRankSum=0.00;POSITIVE_TRAIN_SITE;QD=14.25;ReadPosRankSu
m=-9.000e-02;SOR=0.810;VQSLOD=10.00;culprit=MQRankSum   GT:AD:DP:GQ:PL
      1/1:0,28:28:84:905,84,0
chr19 48744164    .     C     T     8256.82     PASS
      AC=2;AF=1.00;AN=2;BaseQRankSum=0.00;ClippingRankSum=0.00;DP=22;Exce
ssHet=0.0658;FS=2.739;InbreedingCoeff=0.3208;MQ=59.45;MQRankSum=0.00;POSI
TIVE_TRAIN_SITE;QD=17.38;ReadPosRankSum=0.280;SOR=0.974;VQSLOD=7.69;culpr
it=MQRankSum     GT:AD:DP:GQ:PL   1/1:0,22:22:66:635,66,0
chr19 55250939    .     A     G     186.22      PASS
      AC=1;AF=0.500;AN=2;BaseQRankSum=0.00;ClippingRankSum=0.00;DP=7;Exce
ssHet=3.0702;FS=0.000;InbreedingCoeff=-
0.0344;MQ=34.19;MQRankSum=2.19;NEGATIVE_TRAIN_SITE;QD=16.93;ReadPosRankSu
m=1.38;SOR=0.446;VQSLOD=-3.637e+00;culprit=MQRankSum
      GT:AD:DP:GQ:PGT:PID:PL 0/1:3,4:7:99:0|1:55250939_A_G:159,0,132
chr19 55250962    .     T     G     183.15      PASS
      AC=1;AF=0.500;AN=2;BaseQRankSum=0.00;ClippingRankSum=0.00;DP=8;Exce
ssHet=3.0702;FS=0.000;InbreedingCoeff=-
0.0380;MQ=33.52;MQRankSum=2.45;NEGATIVE_TRAIN_SITE;QD=15.26;ReadPosRankSu
m=0.566;SOR=0.693;VQSLOD=-3.672e+00;culprit=MQRankSum
      GT:AD:DP:GQ:PGT:PID:PL 0/1:4,4:8:99:0|1:55250939_A_G:156,0,154
chr19 55250991    .     G     A     62.21 PASS
      AC=1;AF=0.500;AN=2;BaseQRankSum=0.674;ClippingRankSum=0.00;DP=8;Exc
essHet=3.0719;FS=0.000;InbreedingCoeff=-
0.0304;MQ=33.52;MQRankSum=2.45;NEGATIVE_TRAIN_SITE;QD=5.18;ReadPosRankSum
=-1.383e+00;SOR=0.693;VQSLOD=-4.404e+00;culprit=MQRankSum
      GT:AD:DP:GQ:PL   0/1:4,4:8:75:75,0,75
chr20 25301022    .     G     A     8463.11     PASS
      AC=2;AF=1.00;AN=2;BaseQRankSum=-3.110e-
01;ClippingRankSum=0.00;DP=24;ExcessHet=1.7878;FS=0.000;InbreedingCoeff=0
.0519;MQ=60.00;MQRankSum=0.00;POSITIVE_TRAIN_SITE;QD=15.50;ReadPosRankSum
=0.00;SOR=0.663;VQSLOD=15.30;culprit=MQRankSum     GT:AD:DP:GQ:PL
      1/1:0,24:24:72:713,72,0
chr20 60884339    .     G     A     1131.24     PASS
      AC=1;AF=0.500;AN=2;BaseQRankSum=-
1.889e+00;ClippingRankSum=0.00;DP=19;ExcessHet=3.9352;FS=7.249;Inbreeding
Coeff=-
0.0870;MQ=60.00;MQRankSum=0.00;POSITIVE_TRAIN_SITE;QD=13.00;ReadPosRankSu
m=0.152;SOR=0.203;VQSLOD=14.00;culprit=MQRankSum   GT:AD:DP:GQ:PL
      0/1:6,13:19:99:286,0,164
chr20 60889475    .     C     T     1927.25     PASS
      AC=1;AF=0.500;AN=2;BaseQRankSum=-7.360e-
01;ClippingRankSum=0.00;DP=101;ExcessHet=3.0605;FS=1.910;InbreedingCoeff=
-
0.0233;MQ=60.00;MQRankSum=0.00;QD=9.83;ReadPosRankSum=1.47;SOR=0.551;VQSL
OD=13.92;culprit=MQRankSum  GT:AD:DP:GQ:PL   0/1:55,46:101:99:1039,0,1277
chr20 60897768    .     G     A     465.39      PASS
      AC=1;AF=0.500;AN=2;BaseQRankSum=1.91;ClippingRankSum=0.00;DP=17;Exc
essHet=3.0671;FS=1.290;InbreedingCoeff=-
0.0290;MQ=60.00;MQRankSum=0.00;QD=12.25;ReadPosRankSum=-9.250e-
01;SOR=1.085;VQSLOD=14.03;culprit=MQRankSum  GT:AD:DP:GQ:PGT:PID:PL
      0/1:3,14:17:84:0|1:60897768_G_A:373,0,84
chr20 61471799    .     A     T     10863.19    PASS
      AC=2;AF=1.00;AN=2;BaseQRankSum=0.317;ClippingRankSum=0.00;DP=44;Exc
```

essHet=3.5147;FS=1.750;InbreedingCoeff=-
0.0448;MQ=59.97;MQRankSum=0.00;POSITIVE_TRAIN_SITE;QD=12.47;ReadPosRankSu
m=0.609;SOR=0.856;VQSLOD=9.18;culprit=MQRankSum GT:AD:DP:GQ:PL
1/1:0,44:44:99:1374,132,0
chr21 34804966 . T C 15782.70 PASS
AC=2;AF=1.00;AN=2;BaseQRankSum=1.70;ClippingRankSum=0.00;DP=15;Exce
ssHet=4.9489;FS=12.843;InbreedingCoeff=-
0.0931;MQ=59.96;MQRankSum=0.00;POSITIVE_TRAIN_SITE;QD=22.39;ReadPosRankSu
m=0.246;SOR=0.123;VQSLOD=8.63;culprit=MQRankSum GT:AD:DP:GQ:PL
1/1:0,15:15:44:463,44,0
chr21 42808015 . A G 27798.53 PASS
AC=2;AF=1.00;AN=2;BaseQRankSum=-6.680e-
01;ClippingRankSum=0.00;DP=43;ExcessHet=3.3707;FS=0.000;InbreedingCoeff=-
0.0313;MQ=59.98;MQRankSum=0.00;QD=22.47;ReadPosRankSum=-2.120e-
01;SOR=0.687;VQSLOD=9.68;culprit=MQRankSum GT:AD:DP:GQ:PL
1/1:0,43:43:99:1352,129,0
chr21 42808054 . C G 13680.30 PASS
AC=2;AF=1.00;AN=2;BaseQRankSum=-
1.358e+00;ClippingRankSum=0.00;DP=24;ExcessHet=0.7929;FS=0.000;Inbreeding
Coeff=0.0951;MQ=59.96;MQRankSum=0.00;POSITIVE_TRAIN_SITE;QD=22.03;ReadPos
RankSum=0.254;SOR=0.730;VQSLOD=10.39;culprit=MQRankSum GT:AD:DP:GQ:PL
1/1:0,24:24:72:791,72,0
chr21 42817582 . A G 30142.64 PASS
AC=2;AF=1.00;AN=2;BaseQRankSum=2.71;ClippingRankSum=0.00;DP=20;Exce
ssHet=5.8072;FS=3.967;InbreedingCoeff=-
0.1116;MQ=60.00;MQRankSum=0.00;POSITIVE_TRAIN_SITE;QD=17.94;ReadPosRankSu
m=-8.000e-03;SOR=1.180;VQSLOD=14.40;culprit=MQRankSum GT:AD:DP:GQ:PL
1/1:0,20:20:60:676,60,0
chr21 42824874 . C T 12199.36 PASS
AC=2;AF=1.00;AN=2;BaseQRankSum=-5.900e-
01;ClippingRankSum=0.00;DP=16;ExcessHet=2.6749;FS=0.000;InbreedingCoeff=0
.0030;MQ=60.00;MQRankSum=0.00;POSITIVE_TRAIN_SITE;QD=17.73;ReadPosRankSum
=0.891;SOR=0.704;VQSLOD=14.89;culprit=MQRankSum GT:AD:DP:GQ:PL
1/1:0,16:16:48:475,48,0
chr22 17586583 . C G 12967.08 PASS
AC=2;AF=1.00;AN=2;BaseQRankSum=-6.800e-
02;ClippingRankSum=0.00;DP=24;ExcessHet=5.2317;FS=4.297;InbreedingCoeff=-
0.1083;MQ=60.05;MQRankSum=0.00;POSITIVE_TRAIN_SITE;QD=19.50;ReadPosRankSu
m=-2.420e-01;SOR=0.407;VQSLOD=7.80;culprit=MQRankSum GT:AD:DP:GQ:PL
1/1:0,24:24:72:790,72,0
chr22 18975345 . A C 6618.77 PASS
AC=2;AF=1.00;AN=2;BaseQRankSum=2.40;ClippingRankSum=0.00;DP=108;Exc
essHet=0.3915;FS=2.572;InbreedingCoeff=0.2846;MQ=59.78;MQRankSum=0.00;POS
ITIVE_TRAIN_SITE;QD=15.43;ReadPosRankSum=-
1.308e+00;SOR=0.517;VQSLOD=6.30;culprit=MQRankSum GT:AD:DP:GQ:PL
1/1:0,108:108:99:3042,323,0
chr22 18975446 . G C 5849.77 PASS
AC=2;AF=1.00;AN=2;BaseQRankSum=-5.800e-
02;ClippingRankSum=0.00;DP=98;ExcessHet=0.3915;FS=0.439;InbreedingCoeff=0
.2846;MQ=59.66;MQRankSum=0.00;POSITIVE_TRAIN_SITE;QD=16.25;ReadPosRankSum
=0.605;SOR=0.659;VQSLOD=7.21;culprit=MQRankSum GT:AD:DP:GQ:PL
1/1:0,98:98:99:2714,293,0
chr22 18975504 . A C 2436.77 PASS
AC=2;AF=1.00;AN=2;BaseQRankSum=2.24;ClippingRankSum=0.00;DP=33;Exce
ssHet=0.3915;FS=7.120;InbreedingCoeff=0.2842;MQ=60.00;MQRankSum=0.00;POSI
TIVE_TRAIN_SITE;QD=19.19;ReadPosRankSum=0.148;SOR=0.275;VQSLOD=10.39;culp
rit=MQRankSum GT:AD:DP:GQ:PL 1/1:0,33:33:99:982,99,0

chr22 19960184 . C G 12546.39 PASS
AC=2;AF=1.00;AN=2;BaseQRankSum=0.388;ClippingRankSum=0.00;DP=36;Exc
essHet=1.7328;FS=1.130;InbreedingCoeff=0.0452;MQ=60.00;MQRankSum=0.00;POS
ITIVE_TRAIN_SITE;QD=18.84;ReadPosRankSum=0.00;SOR=0.799;VQSLOD=14.06;culp
rit=MQRankSum GT:AD:DP:GQ:PGT:PID:PL
1/1:0,36:36:99:1|1:19960184_C_G:1710,114,0
chr22 19960198 . G C 13892.54 PASS
AC=2;AF=1.00;AN=2;BaseQRankSum=-6.120e-
01;ClippingRankSum=0.00;DP=43;ExcessHet=1.3050;FS=0.000;InbreedingCoeff=0
.0762;MQ=60.00;MQRankSum=0.00;POSITIVE_TRAIN_SITE;QD=16.88;ReadPosRankSum
=0.172;SOR=0.677;VQSLOD=14.31;culprit=MQRankSum GT:AD:DP:GQ:PGT:PID:PL
1/1:0,43:43:99:1|1:19960184_C_G:1854,129,0
chr22 19978022 . C T 8647.38 PASS
AC=2;AF=1.00;AN=2;BaseQRankSum=-5.310e-
01;ClippingRankSum=0.00;DP=14;ExcessHet=2.7327;FS=0.532;InbreedingCoeff=0
.0054;MQ=59.97;MQRankSum=0.00;POSITIVE_TRAIN_SITE;QD=13.37;ReadPosRankSum
=-2.100e-01;SOR=0.625;VQSLOD=10.86;culprit=MQRankSum GT:AD:DP:GQ:PL
1/1:0,14:14:42:445,42,0
chr22 29885594 . A T 960.53 PASS
AC=1;AF=0.500;AN=2;BaseQRankSum=-9.030e-
01;ClippingRankSum=0.00;DP=13;ExcessHet=5.1737;FS=18.056;InbreedingCoeff=
-0.0864;MQ=72.86;MQRankSum=4.57;QD=7.62;ReadPosRankSum=-
2.234e+00;SOR=1.911;VQSLOD=-5.814e+00;culprit=MQRankSum
GT:AD:DP:GQ:PGT:PID:PL 0/1:4,9:13:99:0|1:29885594_A_T:293,0,508
chr22 29885598 . AAGGAAG A 799.84 PASS
AC=1;AF=0.500;AN=2;BaseQRankSum=0.199;ClippingRankSum=0.00;DP=16;Ex
cessHet=3.7468;FS=19.945;InbreedingCoeff=-
0.0539;MQ=62.46;MQRankSum=4.56;NEGATIVE_TRAIN_SITE;QD=10.25;ReadPosRankSu
m=-2.884e+00;SOR=2.273;VQSLOD=-7.867e-01;culprit=ReadPosRankSum
GT:AD:DP:GQ:PGT:PID:PL 0/1:7,9:16:99:0|1:29885594_A_T:290,0,538
chr22 29957911 . T C 13908.51 PASS
AC=2;AF=1.00;AN=2;BaseQRankSum=1.02;ClippingRankSum=0.00;DP=13;Exce
ssHet=0.9975;FS=1.022;InbreedingCoeff=0.1315;MQ=60.00;MQRankSum=0.00;POSI
TIVE_TRAIN_SITE;QD=25.76;ReadPosRankSum=0.010;SOR=0.557;VQSLOD=10.42;culp
rit=MQRankSum GT:AD:DP:GQ:PL 1/1:0,13:13:39:471,39,0
chr22 38536264 . T C 15.21 PASS
AC=1;AF=0.500;AN=2;BaseQRankSum=2.31;ClippingRankSum=0.00;DP=12;Exc
essHet=3.0594;FS=0.000;InbreedingCoeff=-
0.0286;MQ=60.00;MQRankSum=0.00;QD=1.27;ReadPosRankSum=-
1.837e+00;SOR=0.307;VQSLOD=11.35;culprit=MQRankSum GT:AD:DP:GQ:PGT:PID:PL
0/1:10,2:12:54:0|1:38536264_T_C:54,0,591
chr22 38536266 . G A 15.32 PASS
AC=1;AF=0.500;AN=2;BaseQRankSum=0.109;ClippingRankSum=0.00;DP=12;Ex
cessHet=3.0594;FS=0.000;InbreedingCoeff=-
0.0332;MQ=60.00;MQRankSum=0.00;QD=1.28;ReadPosRankSum=-
1.837e+00;SOR=0.307;VQSLOD=11.37;culprit=MQRankSum GT:AD:DP:GQ:PGT:PID:PL
0/1:10,2:12:54:0|1:38536264_T_C:54,0,591
chr22 39497181 . T A 7701.95 PASS
AC=2;AF=1.00;AN=2;BaseQRankSum=0.297;ClippingRankSum=0.00;DP=16;Exc
essHet=3.8153;FS=2.501;InbreedingCoeff=-
0.0697;MQ=60.00;MQRankSum=0.00;POSITIVE_TRAIN_SITE;QD=14.75;ReadPosRankSu
m=-9.350e-01;SOR=0.611;VQSLOD=15.16;culprit=MQRankSum
GT:AD:DP:GQ:PGT:PID:PL 1/1:0,16:16:48:1|1:39497160_C_T:530,48,0
chr22 39498677 . A C 7147.48 PASS
AC=2;AF=1.00;AN=2;BaseQRankSum=0.955;ClippingRankSum=0.00;DP=15;Exc
essHet=5.2774;FS=0.565;InbreedingCoeff=-
0.1085;MQ=58.18;MQRankSum=0.00;POSITIVE_TRAIN_SITE;QD=14.92;ReadPosRankSu

m=-2.530e-01;SOR=0.659;VQSLOD=5.69;culprit=MQRankSum GT:AD:DP:GQ:PL
1/1:0,15:15:45:466,45,0
chr22 39499604 . T C 6511.17 PASS
AC=2;AF=1.00;AN=2;BaseQRankSum=1.14;ClippingRankSum=0.00;DP=11;Exce
ssHet=6.0814;FS=2.499;InbreedingCoeff=-
0.1198;MQ=59.86;MQRankSum=0.00;POSITIVE_TRAIN_SITE;QD=15.32;ReadPosRankSu
m=0.00;SOR=0.534;VQSLOD=7.74;culprit=MQRankSum GT:AD:DP:GQ:PL
1/1:0,11:11:33:397,33,0
chr22 39499763 . T C 18538.60 PASS
AC=2;AF=1.00;AN=2;BaseQRankSum=0.509;ClippingRankSum=0.00;DP=45;Exc
essHet=7.2851;FS=0.000;InbreedingCoeff=-
0.1513;MQ=59.98;MQRankSum=0.00;POSITIVE_TRAIN_SITE;QD=13.91;ReadPosRankSu
m=-5.760e-01;SOR=0.669;VQSLOD=10.73;culprit=MQRankSum
GT:AD:DP:GQ:PGT:PID:PL 1/1:0,45:45:99:1|1:39499756_C_T:1285,135,0
chr22 40827319 . G A 2088.04 PASS
AC=2;AF=1.00;AN=2;BaseQRankSum=-5.660e-
01;ClippingRankSum=0.00;DP=17;ExcessHet=2.4407;FS=1.614;InbreedingCoeff=0
.0060;MQ=60.00;MQRankSum=0.00;POSITIVE_TRAIN_SITE;QD=13.56;ReadPosRankSum
=-4.310e-01;SOR=0.890;VQSLOD=15.76;culprit=MQRankSum GT:AD:DP:GQ:PL
1/1:0,17:17:50:518,50,0
chr22 42524696 . T C 4286.89 PASS
AC=2;AF=1.00;AN=2;BaseQRankSum=0.470;ClippingRankSum=0.00;DP=22;Exc
essHet=0.1846;FS=2.526;InbreedingCoeff=0.2566;MQ=58.33;MQRankSum=0.749;NE
GATIVE_TRAIN_SITE;QD=11.05;ReadPosRankSum=0.446;SOR=0.831;VQSLOD=-9.516e-
01;culprit=MQRankSum GT:AD:DP:GQ:PL 1/1:0,22:22:66:708,66,0
chr22 46762301 . G A 988.31 PASS
AC=1;AF=0.500;AN=2;BaseQRankSum=-2.720e-
01;ClippingRankSum=0.00;DP=47;ExcessHet=3.0605;FS=0.758;InbreedingCoeff=-
0.0254;MQ=60.00;MQRankSum=0.00;QD=9.98;ReadPosRankSum=0.201;SOR=0.564;VQS
LOD=14.17;culprit=MQRankSum GT:AD:DP:GQ:PL 0/1:23,24:47:99:491,0,450
chr22 46774558 . C T 2958.29 PASS
AC=1;AF=0.500;AN=2;BaseQRankSum=-3.540e-
01;ClippingRankSum=0.00;DP=154;ExcessHet=3.0605;FS=0.433;InbreedingCoeff=
-
0.0246;MQ=60.00;MQRankSum=0.00;QD=10.49;ReadPosRankSum=2.49;SOR=0.736;VQS
LOD=13.58;culprit=MQRankSum GT:AD:DP:GQ:PL 0/1:76,78:154:99:1556,0,1490
chr22 51117800 . G A 3382.47 PASS
AC=1;AF=0.500;AN=2;BaseQRankSum=2.94;ClippingRankSum=0.00;DP=102;Ex
cessHet=34.8124;FS=25.854;InbreedingCoeff=-0.4437;MQ=66.01;MQRankSum=-
5.265e+00;QD=2.98;ReadPosRankSum=-4.510e-01;SOR=3.797;VQSLOD=-
8.651e+00;culprit=MQRankSum GT:AD:DP:GQ:PGT:PID:PL
0/1:94,8:102:50:0|1:51117800_G_A:50,0,4340
chr22 51117802 . G T 3378.57 PASS
AC=1;AF=0.500;AN=2;BaseQRankSum=2.04;ClippingRankSum=0.00;DP=102;Ex
cessHet=31.2372;FS=23.444;InbreedingCoeff=-0.4281;MQ=66.14;MQRankSum=-
5.105e+00;QD=3.04;ReadPosRankSum=-2.100e-01;SOR=3.645;VQSLOD=-
7.669e+00;culprit=MQRankSum GT:AD:DP:GQ:PGT:PID:PL
0/1:94,8:102:50:0|1:51117800_G_A:50,0,4340
chr22 51117811 . C T 3061.56 PASS
AC=1;AF=0.500;AN=2;BaseQRankSum=3.90;ClippingRankSum=0.00;DP=101;Ex
cessHet=31.2372;FS=22.982;InbreedingCoeff=-0.4297;MQ=65.77;MQRankSum=-
5.105e+00;QD=2.82;ReadPosRankSum=-3.380e-01;SOR=3.515;VQSLOD=-
7.541e+00;culprit=MQRankSum GT:AD:DP:GQ:PGT:PID:PL
0/1:93,8:101:56:0|1:51117800_G_A:56,0,4242
chr22 51150167 . T C 6398.33 PASS
AC=2;AF=1.00;AN=2;BaseQRankSum=-
2.189e+00;ClippingRankSum=0.00;DP=10;ExcessHet=1.4531;FS=4.992;Inbreeding
Coeff=0.0586;MQ=59.85;MQRankSum=0.00;POSITIVE_TRAIN_SITE;QD=15.88;ReadPos

RankSum=0.330;SOR=0.405;VQSLOD=8.75;culprit=MQRankSum GT:AD:DP:GQ:PL
1/1:0,10:10:30:278,30,0
chrX 47030585 . TGAG T 25.32 PASS
AC=1;AF=0.500;AN=2;BaseQRankSum=-3.570e-
01;ClippingRankSum=0.00;DP=49;ExcessHet=3.0605;FS=1.376;InbreedingCoeff=-
0.0253;MQ=76.36;MQRankSum=0.00;QD=0.42;ReadPosRankSum=0.112;SOR=0.425;VQS
LOD=4.67;culprit=QD GT:AD:DP:GQ:PL 0/1:45,4:49:33:33,0,1830
chrX 53589090 . TTCC T 164.58 PASS
AC=1;AF=0.500;AN=2;BaseQRankSum=-
2.235e+00;ClippingRankSum=0.00;DP=72;ExcessHet=3.0103;FS=1.330;Inbreeding
Coeff=-
0.0115;MQ=60.14;MQRankSum=2.53;NEGATIVE_TRAIN_SITE;QD=2.29;ReadPosRankSum
=1.27;SOR=0.427;VQSLOD=-1.211e+00;culprit=MQRankSum GT:AD:DP:GQ:PL
0/1:62,10:72:99:213,0,2728
chrX 149639324 . ACAG A 1568.08 PASS
AC=1;AF=0.500;AN=2;BaseQRankSum=-8.340e-
01;ClippingRankSum=0.00;DP=99;ExcessHet=6.1017;FS=0.000;InbreedingCoeff=-
0.1429;MQ=59.98;MQRankSum=0.00;QD=1.69;ReadPosRankSum=-3.820e-
01;SOR=0.619;VQSLOD=4.95;culprit=QD GT:AD:DP:GQ:PL
0/1:85,14:99:99:312,0,3555
