# Supplementary figures and images for: Contribution of a Novel B3GLCT Variant to Peters Plus Syndrome Discovered by a Combination of Next-Generation Sequencing and Automated Text Mining
Source: Int J Mol Sci. 2019 Nov 28;20(23):6006. doi: 10.3390/ijms20236006 (PMC6928627; doi:10.3390/ijms20236006)

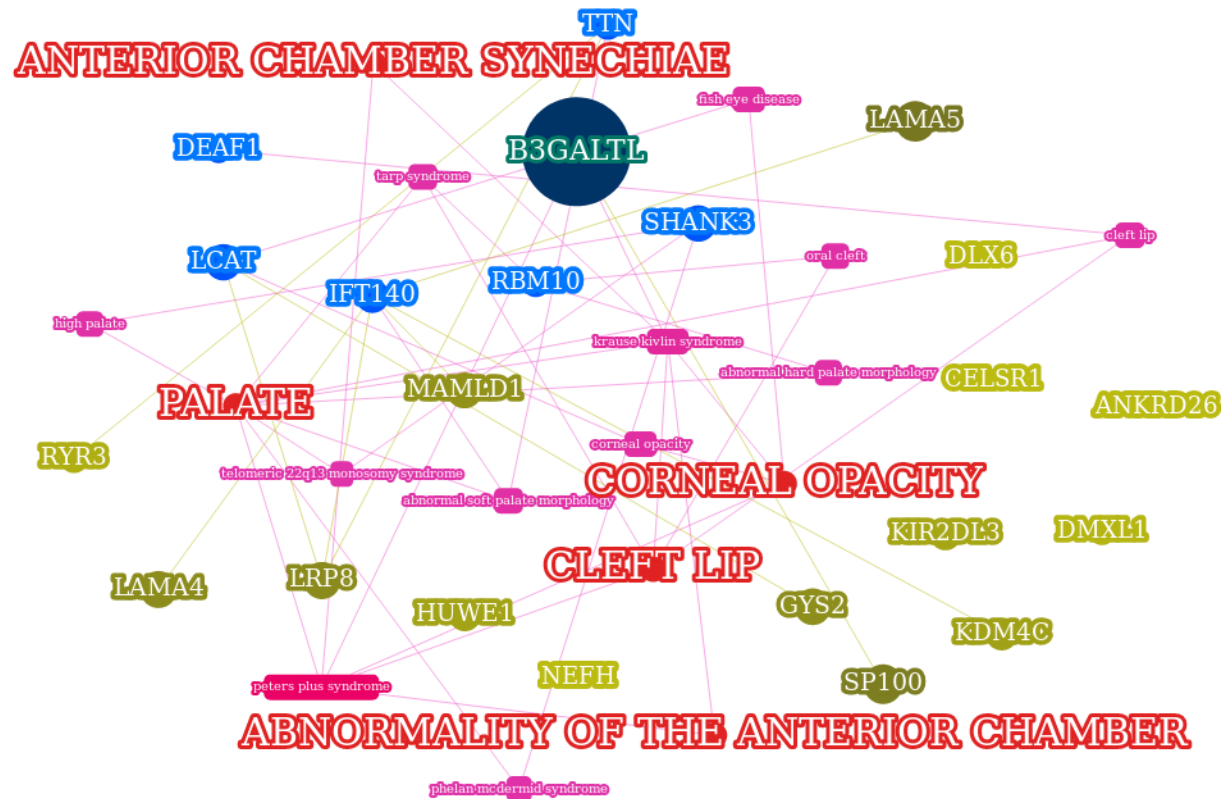

Supplement: Supplementary file 1 [file ijms-20-06006-s001.zip › Toton-Zuranska et al. Supplement_2.pdf]
